# Macrophage-derived EDA-A2 inhibits intestinal stem cells by targeting miR-494/EDA2R/β-catenin signaling in mice

**DOI:** 10.1038/s42003-021-01730-0

**Published:** 2021-02-16

**Authors:** Lele Song, Renxu Chang, Xia Sun, Liying Lu, Han Gao, Huiying Lu, Ritian Lin, Xiaorong Xu, Zhanju Liu, Lixing Zhan

**Affiliations:** 1 CAS Key Laboratory of Nutrition, Metabolism and Food Safety, Shanghai Institute of Nutrition and Health, Shanghai Institutes for Biological Sciences, University of Chinese Academy of Sciences, Chinese Academy of Sciences, Shanghai, China; 2 Department of Gastroenterology, The Shanghai Tenth People’s Hospital, Tongji University, Shanghai, China; 3 Department of Oncology, Changhai Hospital, The Second Military Medical University, Shanghai, China

**Keywords:** Intestinal stem cells, Inflammation, Inflammatory bowel disease

## Abstract

The mucosa microenvironment is critical for intestinal stem cell self-renewal and reconstruction of the epithelial barrier in inflammatory bowel disease (IBD), where the mechanisms underlying cross-talk between intestinal crypts and the microenvironment remain unclear. Here, we firstly identified miR-494-3p as an important protector in colitis. miR-494-3p levels were decreased and negatively correlated with the severity in human IBD samples, as well as in colitis mice. In colitis crypts, a notable cytokine–cytokine receptor, miR-494-3p-targeted EDA2R and the ligand EDA-A2, suppressed colonic stemness and epithelial repair by inhibiting β-catenin/c-Myc. In differentiated IECs, miR-494-3p inhibits macrophage recruitment, M1 activation and EDA-A2 secretion by targeting IKKβ/NF-κB in colitis. A miR-494-3p agomir system notably ameliorated the severity of colonic colitis in vivo. Collectively, our findings uncover a miR-494-3p-mediated cross-talk mechanism by which macrophage-induced intestinal stem cell impairment aggravates intestinal inflammation.

## Abstract (web summary)

Song, Chang et al. identify miR-494-3p as a protector of colitis in mice. They find that a cytokine–cytokine receptor, miR-494-3p-targeted EDA2R and its ligand EDA-A2 derived from macrophages, suppresses colonic stemness and epithelial repair in colitis crypts. This study illustrates how macrophage-induced impairment of intestinal stem cells aggravates intestinal inflammation via miRNA.

## Introduction

The incidence and prevalence of inflammatory bowel disease (IBD), an inflammatory disorder of the gastrointestinal tract that comprises both ulcerative colitis (UC) and Crohn’s disease (CD)^1,2^, is dramatically increasing across the globe in the last three decades^3^. The pathogenesis of IBD is complex, which is contributed by the interactions between the genetic, the abundant immune responses and microbial or environmental risk factor^1,2^. The diagnosis and therapeutic approaches for IBD remain a major unmet medical need. Hence, a better understanding of the mechanisms whereby intestinal homeostasis disruption drives the development of IBD is expected to facilitate the design of more effective therapies.

Intestinal epithelial cells (IECs) play a crucial role in maintaining the intestinal homeostasis^4^, and a breakdown of immune tolerance to microbiota or gut homeostasis drives the development of IBD^2^. To maintain the intestinal barrier integrity for the regeneration of the injured mucosa, the single-layered intestinal epithelium undergoes constant renewal by highly proliferative stem cells at the base of the colonic crypts^5,6^. Widely conserved pathways, particularly Wnt/β-catenin signaling, have been observed to control the expansion of crypts stem cells and subsequent differentiation during tissue injury and repair in IBD^7,8^. Briefly, in the canonical Wnt signaling branch, Wnt family and R-spondin proteins induce the stabilization of the transcription co-factor β-catenin. β-catenin, together with transcription factor/lymphoid enhancer-binding factor (TCF/LEF)-type transcription factors, drives the expression of target genes to promote stem cell identity and cell cycle progression^9,10^. Recent studies using β-catenin/TCF reporter animals demonstrated that the Wnt pathway is activated during tissue repair^9^. Thus, Wnt/β-catenin signaling is a central determinant of stem cell maintenance and proliferation in epithelial homeostasis, and pathway inhibition results in crypt loss and tissue degeneration^7,8,11^.

The mucosal microenvironment has important effects on the reconstruction of the IEC barrier function in IBD^4^. In this context, activated inflammatory signals, particularly toll-like receptors (TLRs)/inhibitor of nuclear factor kappa B kinase subunit beta (IKKβ)/nuclear factor-kappa B (NF-κB), in intestinal epithelium accelerate the recruitment of immune cells and the production of inflammatory cytokines. Those cytokines hijack IECs survival and regeneration and further impair intestinal integrity, leading to a vicious cycle that eventually induces IBD^12^. However, the precise nature of interactions between IECs, specially the crypt stem cells (CSCs), and immune cells in IBD is not completely understood nor is the significance of these interactions for intestinal stem cells^1,4^.

microRNAs (miRs) play a critical role in post-transcriptional regulation via targeting 3′-untranslated regions (UTRs) of candidate genes to dampen translation or to promote mRNAs degradation processes that are directly relevant to diverse inflammatory diseases, including IBD^11,13^. With the first cancer-targeted microRNA drug, MRX34 entering phase I clinical trial in patients with advanced hepatocellular carcinoma, 49 microRNA therapeutics are attracting special attention recently^14,15^. However, less progress has been made in the use of microRNAs in the therapy of IBD by targeting on the function of colonic stemness.

Herein, we identified miR-494-3p as important for the protection of intestinal stem cells, and miR-494-3p both in serum and colonic tissues are negatively correlated with clinical outcomes in IBD patients and with the severity of dextran sodium sulfate (DSS)-induced mice. Mechanistically, miR-494-3p deficiency in colonic crypt stem cells mediated cross-talk between macrophages and intestinal stem cells by activation of the ectodysplasin A/ectodysplasin A2 receptor (EDA-A2/EDA2R) pathway to block Wnt/β-catenin/c-Myc axis, leading to impairment of intestinal stemness. Also, miR-494-3p deficiency in differentiated IECs integrated the colonic epithelium with lamina propria (LP) macrophages via activation of the IKKβ/NF-κB pathway, promoting macrophages recruitment, M1 activation, and EDA-A2 secretion in DSS-induced mouse colitis as well as in cultured macrophages. Both in vivo and in vitro, we confirmed the negative correlation between miR-494-3p and its different targeted genes: *IKKβ* in differentiated IECs and *EDA2R* in colonic crypt cells. Notably, the application of a miR-494-3p agomir dramatically ameliorated the severity of colitis. Taken together, our results reveal that miR-494-3p, with a major impact in intestinal homeostasis and stemness, may be a promising biomarker for diagnosis and/or therapy in the management of IBD.

## Results

### miR-494-3p deficiency correlates to colitis

**Fig. 1:**
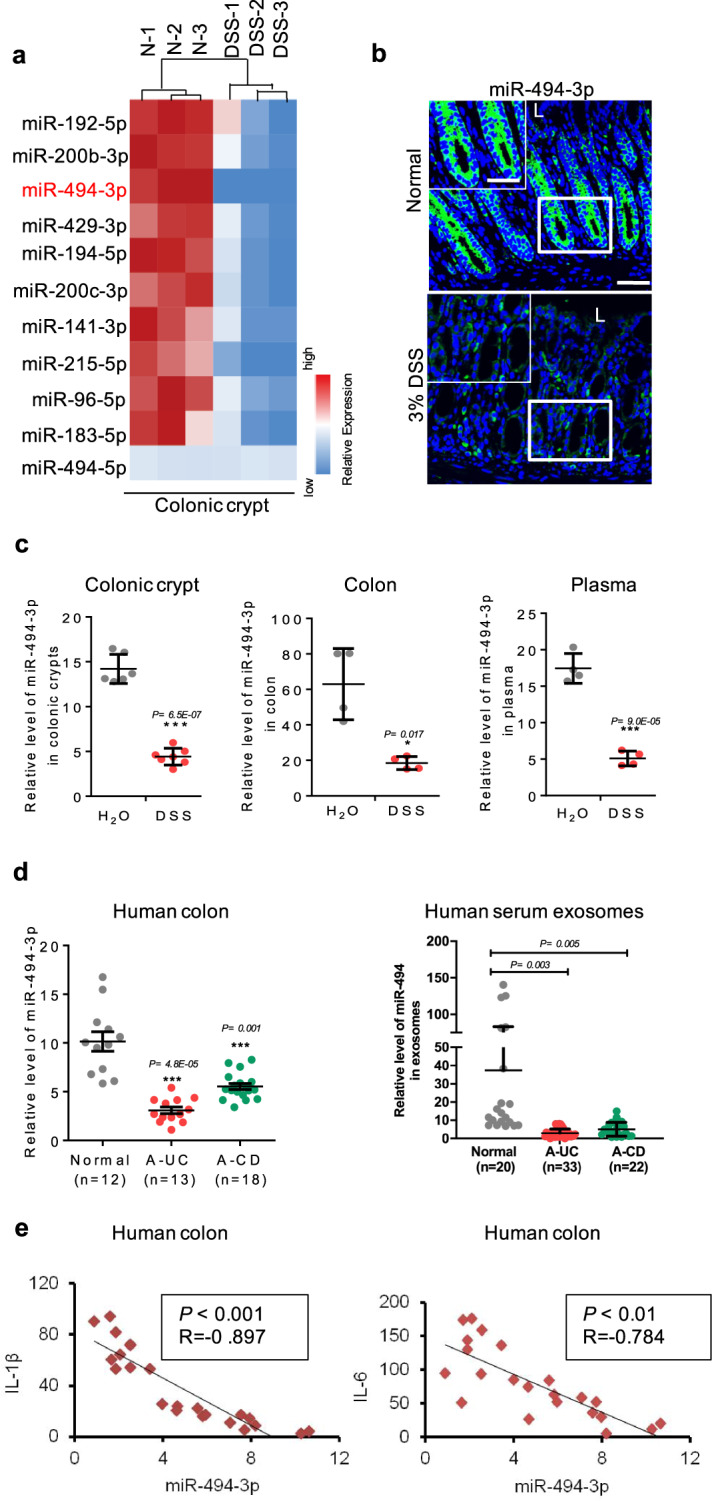
miR-494-3p deficiency relates to colitis. **a**–**c** C57BL/6 mice (male, 8-week-old) were divided into two groups and provided with drinking water neat (Normal, N) or drinking water with 3% DSS (DSS) for 7 days. **a** At day 7, colon crypts were isolated and RNAs were subjected to miRNome analysis. Heat map represented miRs significantly decreased, with ≥2-fold differences and *P* < 0.05 between control group (N) and DSS-treated group (DSS). The color in the heat map is linear with blue as the lowest and red as the highest. **b** miR-494-3p expression and location in colon tissues from RNA-FISH assays, scale bar: 50 μm, scale bar of close-up image: 30 μm. **c** The levels of miR-494-3p in colonic crypts (left), colonic tissues (middle) and plasma (right) were measured by qRT-PCR (normalized by U6 in colon tissue and colonic crypts, normalized by cel-miR-39 in plasma). **d** The levels of miR-494-3p in human colon tissues (left, normalized by U6) and exosomes extracted from human peripheral blood (right, normalized by cel-miR-39). Normal, healthy subject; A-UC, active ulcerative colitis; A-CD, active Crohn’s disease. **e** The plot showed the relationship between miR-494-3p level and IL-1β (left) level or IL-6 (right) level in colon tissues from patients with active IBD (UC and CD). Data are presented as mean ± S.D. and analyzed by non-paired two-tailed Student’s *t-*test in the graphs in (**c**, **d**). ***P ≤ 0.001, **P ≤ 0.01, *P ≤ 0.05.

To identify potential unique miRs that contribute to shaping the intestinal stemness in colitis, we analyzed the miRNome of colonic crypt stem cells from DSS-induced colitis mice. Specifically, we explored whether any overlap could be found between our microarray data and a previously published dataset of colonic tissues from a colitis model^16^. As shown in the both heat maps, a strongly conserved miRs (Supplementary Fig. 1b), miR-494-3p, were decreasing in colonic crypts (compared to normal tissues) (Fig. 1a, Supplementary Fig. 1a). To confirm the change of miR-494-3p and its localization, RNA fluorescence in situ hybridization (RNA-FISH) assay was performed, in which miR-494-3p localized at whole colonic epithelium, especially in colonic crypts, and the levels of miR-494-3p were decreased in both villi and colonic crypts of DSS-induced colitis (Fig. 1b). We further used quantitative real-time PCR (qRT-PCR) to verify the downregulated miRNA-494-3p in both colonic tissues and crypt stem cells (Fig. 1c, Supplementary Fig. 1c), consistent with the results obtained from the dataset analysis (Supplementary Fig. 1d). Moreover, miR-494-3p levels also were declined in the plasma of DSS-treated mice compared to those in normal mice (Fig. 1c). Decrease of miR-494-5p levels in 3% DSS-treated mice was lower than the change of miR-494-3p and did not reach statistical significance (Fig. 1a, Supplementary Fig. 1c). Thus, our data indicate that miR-494-3p but not miR-494-5p may play a role in the pathogenesis of DSS-induced colitis.

To further assess the clinical relevance of miR-494-3p to the pathogenesis of IBD, we examined miR-494-3p levels in samples from active IBD patients. These analyses indicated depletion of miR-494-3p in samples (tissues and serum) from active IBD patients compared to that from healthy subjects, consistent with the analysis from GEO datasets (Fig. 1d, Supplementary Fig. 1e). Additionally, we observed a dramatically negative correlation between the levels of miR-494-3p and pro-inflammatory cytokines interleukin (IL)−1β and IL-6 in active IBD patients (Fig. 1e). Taken together, these results suggest that miR-494-3p depletion is positively associated with the pathogenesis of IBD.

### *miR-494*^−/−^ mice exhibit abnormalities in crypt stem cell identity in DSS-induced colitis

**Fig. 2:**
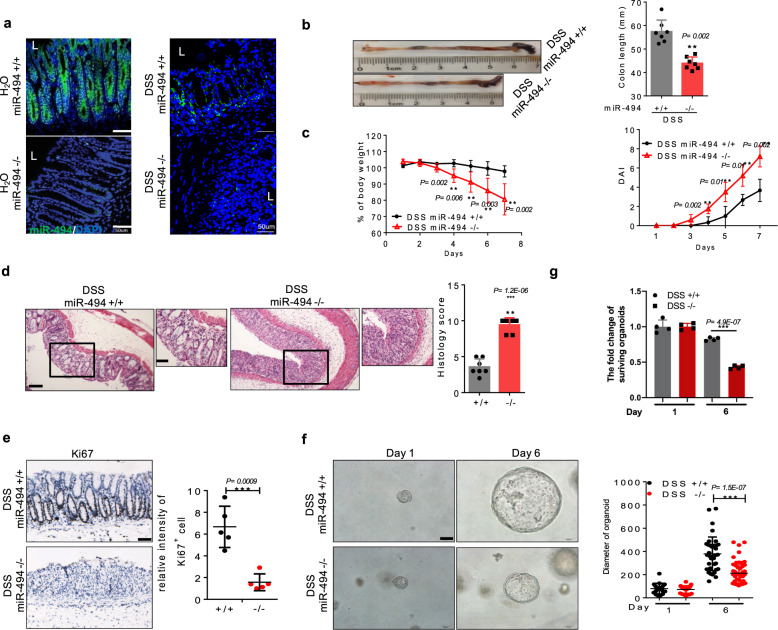
*miR-494*^−/−^ mice exhibit abnormalities in crypt stem cell identity in DSS-induced colitis. **a**–**g** *miR-494* knockout mice (*miR-494*^−/−^, −/−) and wide-type mice (WT, *miR-494*^+/+^, +/+) at 8-weeks old were provided with free access to drinking water with 3% DSS for 7 days. Day 0 is the initiation of DSS treatment. **a** Representative images of miR-494-3p expression in colon tissue from RNA-FISH assay, scale bar: 50 μm. **b** Typical images of the colon from *miR-494*^−/−^ mice and WT mice on day 7. Colon length was measured on day 7 after killing. **c** Body weight changes and DAI. **d** Hematoxylin and eosin (H&E) images of colon tissue and histology score (0–12), scale bar: 200 μm. The scale bar of close-up image is 100 μm. **e** IHC was performed for the proliferation marker Ki67 in colon, scale bar: 100 μm. **f** Representative images of colon organoids derived from colonic crypt that were isolated from DSS-treated mice (on day 7) and then cultured in vitro for 6 days (right), scale bar: 100 μm. **e**, **f** The diameter of organoids and number of Ki67^+^ cells were measured by ImageJ. **g** The quantification of surviving organoids. Scale bar: 100 μm. Data are presented as mean ± S.D. and analyzed by non-paired two-tailed Student’s *t-*test in panels (**b**–**e**). ***P ≤ 0.001, **P ≤ 0.01, *P ≤ 0.05.

To determine the role of miR-494-3p in the pathogenesis of colitis, we generated *miR-494* knockout (*miR-494*^−/−^) mice. The depletion of miR-494-3p in colonic tissues was confirmed using RNA-FISH and qRT-PCR (Fig. 2a, Supplementary Fig. 2a). Under the steady-state conditions, *miR-494*^−/−^ mice displayed normal body weight, intestinal histology and intestinal permeability (Supplementary Fig. 2b–e). Next, we challenged mice with free access to 3% DSS in the drinking water for 7 days. We observed that *miR-494*^−/−^ mice exhibited attenuation of body weight and colon length, and elevation of the disease activity index (DAI) compared to wild-type (WT, *miR-494*^+/+^) mice (Fig. 2b and c). Further histopathological examination revealed that miR-494 deficiency caused extensive epithelial denudation as well as increase in the number of ulcerations and infiltrated immune cells in the colon and more severe crypt loss (Fig. 2d). Consistent with these observations, we found that intestinal permeability was increased in *miR-494*^−/−^ mice (Supplementary Fig. 2f–h).

The steady cycle of apoptosis and regeneration of intestinal epithelial cells is critical to colon self-renewal and repair and becomes extremely important in colitis^6^. Immunohistochemistry (IHC) staining revealed that Ki67^+^-proliferating cells were diminished in *miR-494*^−/−^ mice compared to those in WT mice (Fig. 2e). Notably, we used an intestinal stem cell-derived organoid culture system and observed that colonic crypt cells isolated from WT mice readily developed more organoids than did those from *miR-494*^−/−^ mice (Figs. 2f and g). Additionally, the data from terminal deoxynucleotidyl transferase dUTP nick end labeling (TUNEL) assays further demonstrated that the colons of *miR-494*^−/−^ mice contained higher numbers apoptotic cells than did those of WT animals (Supplementary Fig. 2i), which indicated the increased injury of colonic mucosa in *miR-494*^−/−^ mice. Thus, our results indicate that the loss of miR-494 positively correlates with experimental colitis, a model in which miR-494-related self-renewal of epithelial stem cells maybe essential for the regenerative response of intestine.

### EDA-A2/EDA2R, is identified as attenuating crypt stem cell proliferation in DSS-induced colitis

**Fig. 3:**
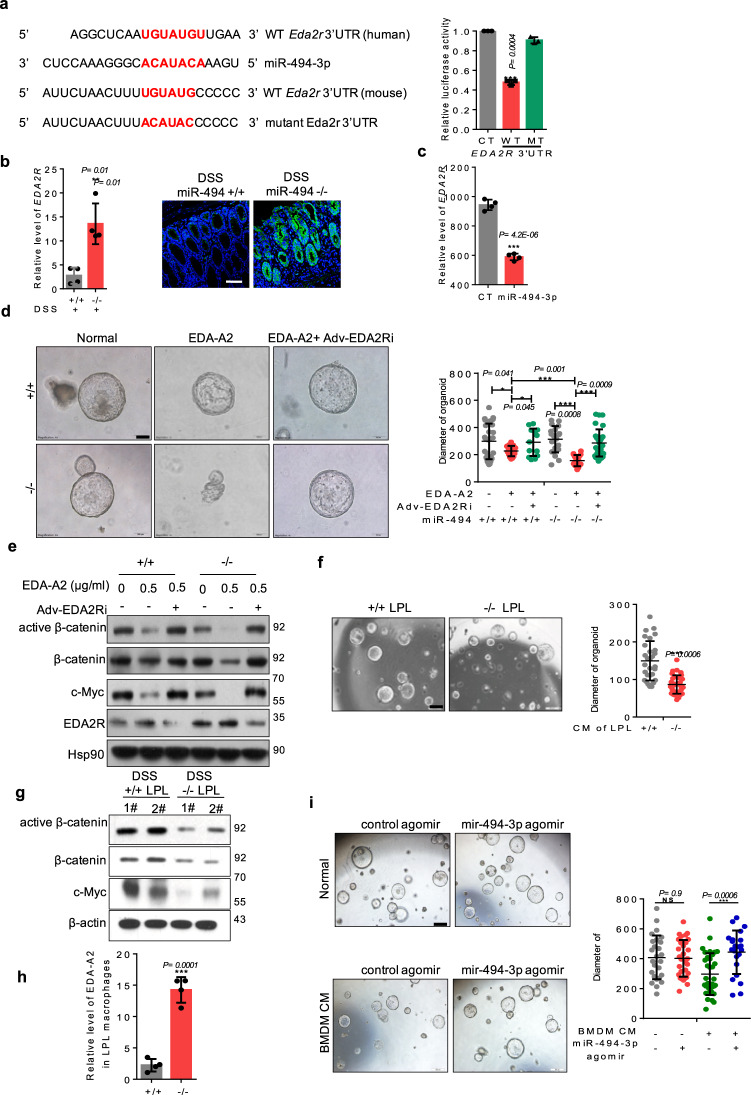
EDA-A2/EDA2R, is identified as attenuating crypt stem cell proliferation in DSS-induced colitis. **a** Sequences of mouse and human *EDA2R* 3′-untranslated regions (UTRs) and conservative miR-494-3p-binding sites. Luciferase activity of the reporter vector containing the WT or mutant 3′-UTR of *EDA2R* was determined after co-transfection with control or miR-494-3p-mimic. **b**, **c** qRT-PCR and IF analysis were performed to measure the levels of EDA2R mRNA (normalized for β-actin) and protein level in colon tissues of DSS-treated WT (+/+) and DSS-treated *miR-494*^−/−^ mice (**b**) or in colonic crypts with miR-494-3p agomir and control agomir incubation (**c**). **d** Representative images and quantification of the size of mice colonic organoids. Organoids derived from the colonic crypts from *miR-494*^−/−^ mice and WT (+/+) mice were incubated with indicated treatment for 5 days, scale bar: 100 μm. **e** Western blot analyses were used to determine the signaling of colonic organoids (**d**). **f** Representative images and quantification of the size of mouse colonic organoids which were cultured with the CM (supernatant of LPL: growth culture medium = 1:1 for 5 days), scale bar: 500 μm. **g** Western blot was used to determine the signaling of colonic organoids (**d**). **h** Levels of *EDA-A2* in LPL macrophages were determined by qRT-PCR (normalized by β-actin). LPL macrophages were isolated from mice provided with free access to drinking water with 3% DSS for 7 days. **i** Representative images and quantification of the size of mice colonic organoids which were incubated with miR-494-3p agomir and control agomir were cultured for 5 days with CM [medium of BMDM (with or without IL-1β and IL-6 treatment): growth culture medium = 1:1]. Scale bar: 500 μm. Data are presented as mean ± S.D. and analyzed by non-paired two-tailed Student’s *t-*test in panels (**a**–**d**, **f**, **h**, **i**). ***P ≤ 0.001; **P ≤ 0.01; *P ≤ 0.05; NS, no significance.

The preservation of intestinal crypt stemness is critical to intestinal self-renewal and epithelial integrity^5,7^. To address the loss of miR-494-dampened proliferation of colonic organoids, we conducted transcriptome profiling using crypts isolated from DSS-treated mice. The statistics of pathway enrichment revealed that the most strongly affected signaling was that corresponding to cytokine–cytokine receptor interactions (Supplementary Fig. 3a). This observation suggested that the altered proliferation of colonic organoids might be modulated by cytokines from the microenvironment and the corresponding receptors in crypts. As shown in a heat map, crypts isolated from DSS-treated *miR-494*^−/−^ mice accumulated higher levels of transcripts encoding cytokine receptors (Supplementary Fig. 3b). When examining the genes encoding these receptors, we noted the presence, in both human and mouse, of a putative miR-494-3p binding site (Fig. 3a) within the 3′ UTR of *EDA2R*, a member of the tumor necrosis factor receptor superfamily^17^. A luciferase reporter assay further confirmed that miR-494-3p directly targeted the *EDA2R* 3′ UTR (Fig. 3a). Furthermore, a negative relationship between miR-494-3p and EDA2R mRNA and protein levels was identified in colon tissues and colonic crypts using qRT-PCR and IF assays (Figs. 3b and c).

Given that EDA-A2, as the specific ligand of EDA2R, is essential for EDA2R activation and stability^18^, we hypothesized that EDA-A2 from the colonic microenvironment might be indispensable for the loss of miR-494-3p mediated inhibition of organoid proliferation and for the activation of EDA2R signaling in colonic crypts. Thus, we added EDA-A2 to the organoid growth medium and then observed that the EDA-A2 inhibited organoid growth; in this context, miR-494 deficiency further inhibited organoid expansion (Fig. 3d), which mimicked the effect of miR-494 deficiency on the proliferation of colonic crypt stem cells in vivo. However, this effect was counteracted by adenovirus-mediated silencing of *EDA2R* (using Adv-EDA2Ri) (Fig. 3d). Contrastively, ectopic expression of miR-494-3p counteracted the inhibition of proliferation induced by exogenous EDA-A2 (Supplementary Fig. 3c). Previous work has shown that Wnt/β-catenin signaling is critical for intestinal stem cells’ self-renewal^5,19^, which led us to assess whether miR-494-3p correlated with Wnt/β-catenin pathway. The results from western blot and qRT-PCR indicated that the activation of β-catenin/c-Myc and the levels of stemess-associated genes were decreased in colonic crypt cells of DSS-treated *miR-494*^−/−^ mice compared to those from DSS-treated WT mice (Supplementary Fig. 3d). Consistently, miR-494-3p agomir yielded increased accumulation of the transcripts of multiple proliferation- and stemness-related genes in colonic organoids derived from DSS-challenged mice (Supplementary Fig. 3e). Furthermore, exogenous Wnt3a did not accelerate the growth of organoids derived from DSS-treated *miR494*^−/−^ mice (Supplementary Fig. 3f). We further examined the β-catenin/c-Myc signaling in organoids grown in the presence of exogenous EDA-A2. As expected, miR-494 deficiency potentiated EDA-A2-induced inhibition of β-catenin/c-Myc expression; this effect was reversed by Adv-EDA2Ri (Fig. 3e). Interestingly, miR-494-3p had no effect on colonic organoid proliferation under normal conditions (Fig. 3d), which indicate that the EDA-A2/EDA2R signaling is critical for inflammatory state. Altogether, activation of EDA-A2/EDA2R signaling suppresses colonic crypt cell proliferation in DSS-treated *miR-494*^−/−^ mice, an effect mediated via abrogation of the Wnt3a/β-catenin/c-Myc signaling in IBD.

Next, we explored the source of EDA-A2 in *miR-494*^−/−^ mice. Our results showed that *EDA-A2*-encoding transcript accumulated to higher levels in colon tissues, particularly in LP lymphocytes (LPLs) but not in IECs, from DSS-treated *miR-494*^−/−^ mice compared to those in colon tissues from WT mice (Supplementary Fig. 3h), suggesting that LPLs were the main source of EDA-A2 that inhibited the proliferation of colonic crypt stem cells in colitis. To further verify this hypothesis, we collected supernatants from cultures of LPLs of DSS-treated *miR-494*^−/−^ mice and WT mice, respectively. Specifically, colonic organoids were cultured with conditioned medium (CM, consisting of a 1:1 (vol/vol) mixture of growth medium: LPLs medium). As expected, exposure of organoids to CM from LPLs of DSS-treated *miR-494*^−/−^ mice yielded decrease of both organoid growth and β-catenin/c-Myc signaling, compared those in organoids grown in CM from LPLs of DSS-treated WT mice (Fig. 3f, g). We extended this analysis by isolating various immune cells from LP and then assessing the accumulation of *EDA-A2*-encoding transcripts. The data showed that only LP macrophages from DSS-treated *miR-494*^−/−^ mice exhibited higher EDA-A2 mRNA levels than other immune cells in the LP of WT mice (Fig. 3h, Supplementary Fig. 3h). Additionally, incubation of bone marrow-derived macrophages (BMDMs) with IL-1β or IL-6 yielded elevated EDA-A2 mRNA level (Supplementary Fig. 3i), indicating that pro-inflammatory signaling from colonic tissues including IECs may stimulate macrophages to secrete EDA-A2. Functionally, the administration of miR-494-3p agomir counteracted the inhibition of organoids growth which was observed in organoids incubated with CM from BMDMs pre-treated with IL-6 and IL-1β (Fig. 3i).

Together, these results indicate that LP macrophages-derived EDA-A2 dampens crypt stem cells proliferation by activating miR-494-3p deficiency/EDA2R, leading to inhibition of the β-catenin/c-Myc axis in an inflammatory setting, in which EDA-A2 secretion were mediated by IL-1β and IL-6.

### LP macrophages contribute to the increased inflammation observed in DSS-treated *miR-494*^*−/−*^ mice

**Fig. 4:**
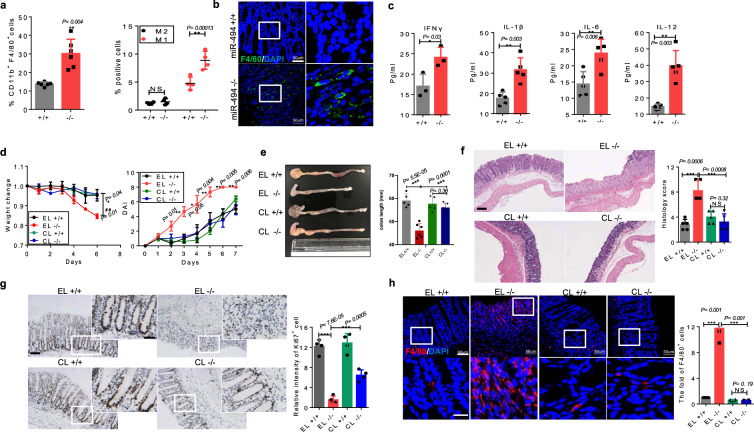
LP macrophages contribute to increased inflammation of DSS-treated *miR-494*^−/−^ mice. **a** Subpopulations of colonic LP macrophage (gated for CD11b^+^CD11C^-^F4/80^+^ cells), M1 (gated for CD11b^+^CD11C^-^F4/80^+^CD206^−^ cells) and M2 (gated for CD11b^+^CD11C^−^F4/80^+^CD206^+^ cells) subsets were analyzed from *miR-494*^−/−^ and WT (+/+) mice on day 7 after DSS treatment. **b** IF staining of colon tissues for macrophage marker F4/80 on day 7 after DSS treatment. Scale bar: 50 μm. **c** The protein levels of each indicated cytokines were determined by ELISA in plasma from *miR-494*^−/−^ mice and WT mice on day 7 after DSS treatment. **d**–**h** Mice were provided with free access to 3% DSS in drinking water for 7 days, and empty liposomes (EL) or clodronate liposomes (CL) was administrated by ip (200 μl/mice) at days −4, 0, 2, 4, and 6. Day 0 is the initial time point of DSS treatment. **d** Body weight changes and DAI of the indicated *miR-494*^−/−^ mice and WT mice after DSS treatment in macrophage deletion assay. **e** Colon length was measured on day 7 after DSS treatment. **f** Typical images of H&E staining of colon tissues, and histological score (0–12), scale bar: 200 μm. **g** IHC staining of colon tissues on day 7 after DSS treatment for proliferation marker Ki67 and the quantification of Ki67^+^cells. Scale bar: 100 μm, scale bar of close-up image: 50 μm. **h** IF staining of colon tissues on day 7 after DSS treatment for macrophage marker F4/80 (scale bar: 50 μm, scale bar of close-up image: 10 μm) and quantification of the number of F4/80^+^ cells. Data are presented as mean ± S.D. and analyzed by non-paired two-tailed Student’s *t-*test in the graphs in (**a**, **c**, **d**–**h**). ***P ≤ 0.001; **P ≤ 0.01; *P ≤ 0.05; NS, no significance.

Given the critical role of macrophage-derived EDA-A2 in colonic stem cell proliferation, we supposed that macrophages might be involved in the increased damage seen in colitis from *miR-494*^−/−^ mice. To investigate the cellular basis for inflammatory responses observed within the colon of *miR-494*^−/−^ mice, we analyzed multiple immune cells in DSS-treated mice using flow cytometry. The number of macrophages, particularly M1 macrophages, was elevated in both colon and spleen of DSS-treated *miR-494*^−/−^ mice compared to that in the corresponding tissues of DSS-treated WT mice (Fig. 4a, Supplementary Fig. 4a), indicating increased recruitment of macrophages in colon of *miR-494*^−/−^ mice. This finding was further confirmed by the results from immunofluorescence (IF) staining for F4/80 (Fig. 4b). Consistent with these results, the levels of pro-inflammatory cytokines (IL-1β, IL-6, IL-12, and IFN-γ) were elevated in the plasma, colon and colonic macrophages of DSS-treated *miR-494*^−/−^ mice compared to those in DSS-treated WT mice (Fig. 3c, Supplementary Fig. 4b and c).

Clodronate liposome (CL) injection is an effective way to specifically deplete macrophages in vivo^20^. Thus, we used administered CLs by intraperitoneal (ip) injection to delete colonic macrophages. The efficiency of macrophage depletion was confirmed by F4/80 staining (Fig. 4h). The results showed that depletion of colonic LP macrophages ameliorated the pathological characteristics of DSS-induced colitis in *miR-494*^−/−^ mice compared with *miR-494*^−/−^ mice treated with empty liposomes (EL) (Fig. 4d–h, Supplementary Fig. 4d–f). Notably, our results demonstrated that local depletion of macrophages specifically abrogated EDA-A2 secretion (Supplementary Fig. 4e). Collectively, these data demonstrate that LP macrophages are necessary to pro-inflammatory response in DSS-treated *miR-494*^−/−^ mice.

### Depletion of miR-494-3p in differentiated IECs promotes macrophage recruitment and M1 polarization in colitis

**Fig. 5:**
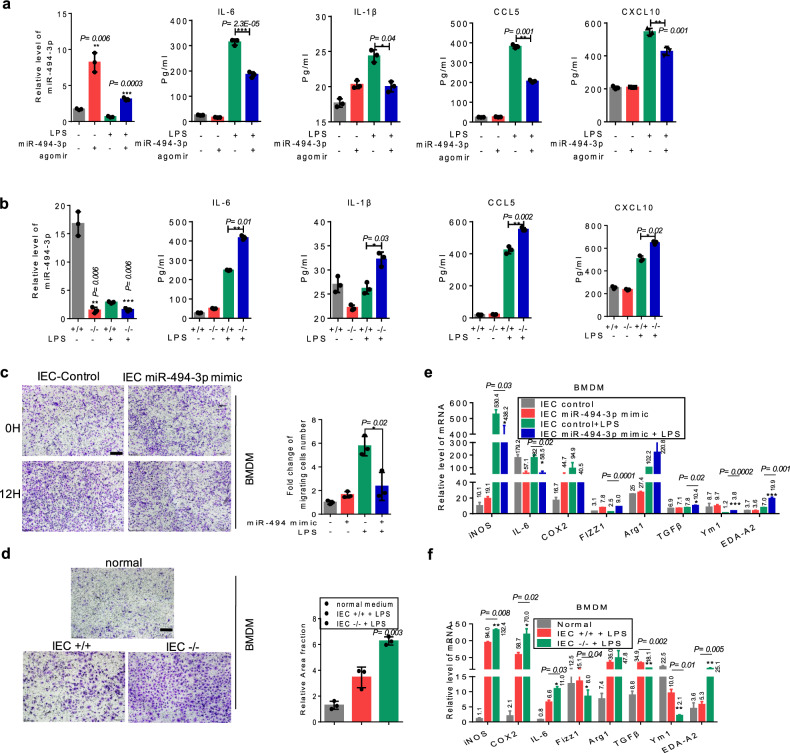
Depletion of miR-494-3p in differentiated IECs promotes macrophage recruitment and M1 polarization in colitis. **a**, **b** The protein levels of each indicated cytokines and chemokines were determined by ELISA in the supernatants of IECs (from WT mice) with or without LPS treatment following control mimic or miR-494 mimic transfection (**a**) or of IECs from *miR-494*^−/−^ mice and WT mice treated with or without LPS (**b**). The levels of miR-494-3p were measured by qRT-PCR (normalized by U6). **c**, **d** Representative images and quantification of the fold change of migrating BMDMs following incubation with CM from indicated IECs that had been transfected with control mimic or miR-494-3p mimic for 24 h (**c**) or from IECs extracted from *miR-494*^−/−^ mice or WT mice (**d**). Scale bar: 200 μm. **e, f** The mRNA levels of M1 and M2 markers were measured by qRT-PCR analysis in BMDMs incubated with CM from IECs with indicated treatment (**e**) or incubated with CM from indicated IECs from *miR-494*^*−*/−^ mice or WT mice (**f**). Except as indicated, all qRT-PCR values were normalized against the expression of the transcript encoding *β-actin*. Data are presented as mean ± S.D. and analyzed by non-paired two-tailed Student’s *t-*test in the graphs in (**a**–**f**). ***P ≤ 0.001, **P ≤ 0.01, *P ≤ 0.05.

Next, we assessed the role of miR-494-3p in the polarization of macrophages. Macrophage-intrinsic miR-494-3p had no effect on macrophage polarization in vitro (Supplementary Fig. 5a, b). Our RNA-FISH results (Fig. 2a) implied that miR-494-3p in IECs affected colonic macrophage functions by regulating the secretion of macrophage-related factors. Therefore, we performed transcriptome profiling on primary colonic IECs isolated from WT mice, induced to be differentiated IECs, transfected with miR-494-3p mimic or control mimic, and subjected to LPS treatment for 24 h. A heat map presented that multiple macrophage-related chemokines were decreased in differentiated IECs with ectopic miR-494-3p (Supplementary Fig. 5c*)*. In particular, the results from enzyme-linked immunosorbent assay (ELISA) and qRT-PCR assays verified that differentiated IECs with ectopic miR-494-3p exhibited lower secretion of pro-inflammatory cytokines (IL-1β, IL-6) and macrophage-related chemokines (Ccl5/6 and Cxcl10/12) (Fig. 5a, Supplementary Fig. 5c, d). We next used chemotaxis assays to investigate the role of differentiated IECs in macrophage recruitment. Notably, supernatants from LPS-treated and differentiated IECs transfected with miR-494-3p mimic inhibited the migration of BMDM cells and RAW264.7 cells compared to LPS-treated IECs transfected with control mimic (Fig. 5c, Supplementary Fig. 5e). Consistent with these findings, deficiency of miR-494-3p in LPS-treated and differentiated IECs yielded notable increase in the expression of pro-inflammatory cytokines (IL-1β, IL-6) and macrophage-related chemokines (Ccl5/6 and Cxcl10/12) (Fig. 5b, Supplementary Fig. 5f) and macrophages’ migration (Fig. 5d).

To clarify whether the miR-494 in differentiated IECs impacted macrophage polarization, M1 and M2 markers were assessed. We noted that BMDMs or RAW264.7 cells were incubated with the medium from LPS-treated and differentiated IECs with ectopic miR-494-3p expression and suppressed M1 polarization (Fig. 5e, Supplementary Fig. 5e). In agreement with this observation, supernatants from LPS-treated and differentiated *miR-494*^−/−^ IECs promoted M1 marker expression in BMDMs (Fig. 5f)

Together, these results demonstrate that loss of miR-494 in differentiated IECs contributes to macrophages recruitment and M1 polarization under inflammatory condition.

### miR-494-3p deficiency in differentiated IECs contributes to colitis via directly regulating IKKβ

**Fig. 6:**
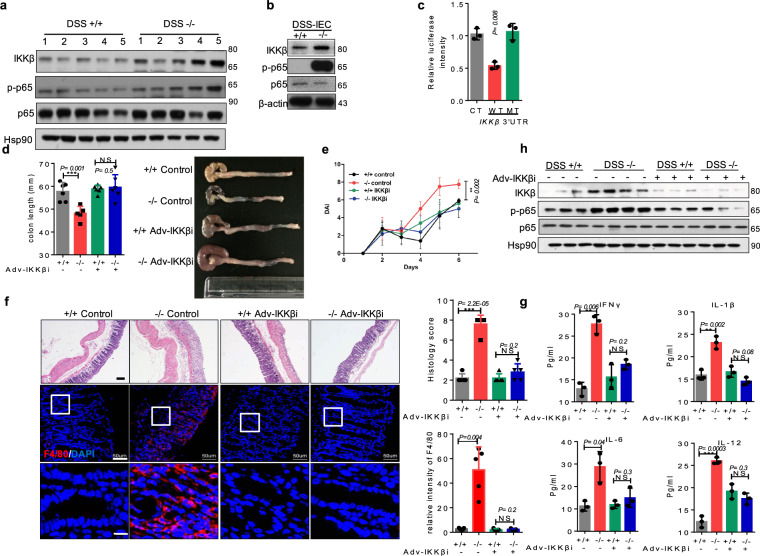
miR-494-3p deficiency in differentiated IECs contributes to colitis via directly regulating IKKβ. **a**, **b** Western blot of colon tissues (**a**) and IECs (**b**) from DSS-treated *miR-494*^−/−^ mice or WT (+/+) mice on day 7. **c** Luciferase activity of the reporter vector containing the sequence of WT or mutant 3′-UTR of *IKKβ* was determined after co-transfection with control or miR-494-3p mimic. **d**–**h** *IKKβ* interference adenovirus (Adv-IKKβi) or control adenovirus was administered to *miR-494*^−/−^ mice and WT (+/+) mice by coloclysis and mice then were provided with free access to 3% DSS in drinking water for 7 days. The number of each group = 5. **d** Colon length was measured on day 7 after killing. **e** The plot showing DAI. **f** Images of H&E (scale bar: 200 μm) and IF staining for macrophage marker F4/80 (scale bar: 50 μm, scale bar of close-up image: 5 μm) in colon tissues at day 7; the right two plots showing histology score (0–12) (upper panel) and quantification of the number of F4/80^+^ cells (lower panel). **g** Levels of indicated cytokines were determined by ELISA in plasma at day 7. **h** Western Blot of colon tissues at day 7. Data are presented as mean ± S.D. and analyzed by non-paired two-tailed Student’s *t-*test in the graphs in (**a**, **d**–**g**). ***P ≤ 0.001; **P ≤ 0.01; *P ≤ 0.05; NS, no significance.

Prior studies demonstrated that IKKβ/NF-κB signaling is excessively activated in the IECs and macrophages of IBD patients, a pattern that is accompanied by increases in the levels of pro-inflammatory cytokines^12^. Consistent with that, we found that loss of miR-494 yielded apparent increase in IKKβ/NF-κB signaling in colonic tissues and differentiated IECs from DSS-treated mice (Fig. 6a, b). To explore the mechanisms, several database predicting tools (miBase, TargetScan, miRDB, and miR.org) were used to identify the putative miR-494-3p-binding sites. A potential site was noted in the 3′-UTR of *IKKβ* (Supplementary Fig. 6a). Using a luciferase reporter assay, we confirmed that only the inclusion of this 3′-UTR, but not the mutant version, impeded the expression of a luciferase-encoding construct, indicating that miR-494-3p directly targeted *IKKβ* 3′-UTR (Fig. 6c).

To further investigate whether miR-494-3p targeted-*IKKβ* regulated DSS-induced colitis, we administered *IKKβ* interference adenovirus (Adv-IKKβi) to downregulate *IKKβ* in the colon. The efficiency of *IKKβ* knockdown (KD) in the colon was verified by western blot (Fig. 6h). We observed that infection of Adv-IKKβi ameliorated DSS-induced colitis in *miR-494*^−/−^ mice (Fig. 6d–f, Supplementary Fig. 6b). These results indicated that IKKβ plays a vital role in the aggravation of colitis present in miR-494 deficiency. IF staining confirmed this inference by showing that the colonic tissues of Adv-IKKβi-*miR494*^−/−^ had increased Ki67^+^ cells and decreased apoptosis cells compared to control *miR-494*^−/−^ mice (Supplementary Fig. 6c). We further revealed that *IKKβ* KD suppressed F4/80^+^ macrophage recruitment, the production of pro-inflammatory cytokines and macrophage-related chemokines, and miR-494 loss-stimulated NF-κB signaling in the colon of DSS-treated *miR-494*^−/−^ mice (Fig. 6f, g, Supplementary Fig. 6d). Additionally, western blot analysis revealed that IKKβ deficiency suppressed miR-494 loss-stimulated NF-κB signaling in mice with DSS-induced colitis (Fig. 6h).

Taken together, these data demonstrated that miR-494-3p deficiency in differentiated IECs activates IKKβ/NF-κB signaling, yielding accumulation of macrophage and potentiation of DSS-induced colitis.

### Administration of miR-494-3p agomir protects mice from colitis

**Fig. 7:**
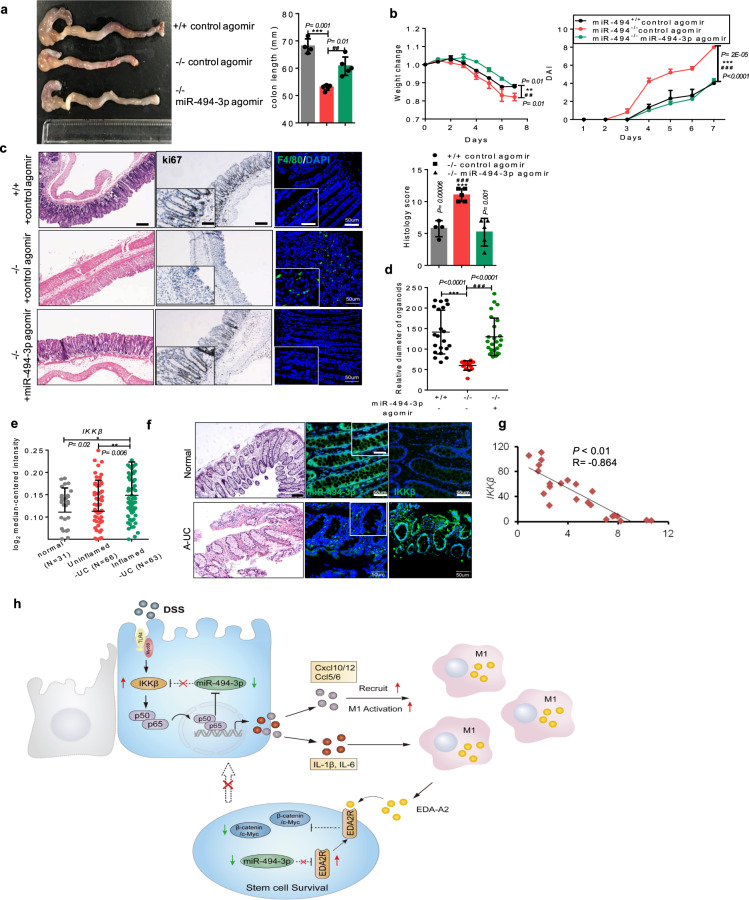
Administration of miR-494-3p agomir protects mice from colitis. **a**–**d** *miR-494*^−/−^ mice and WT (+/+) mice at 8-weeks old were provided with free access to 3% DSS in the drinking water for 7 days. miR-494-3p agomir or control agomir was administered by intravenous at days 0, 2, and 4 (where day 0 is the initial time point of DSS treatment). **a** Typical images of the colon and statistical plot of colon length which was measured at day 7 after sacrifice. **b** Body weight changes and DAI. **c** H&E images of colon tissue (scale bar: 200 μm) and histology score (0–12). IHC staining of colon tissues for proliferation marker Ki67 (scale bar: 200 μm, scale bar of close-up image: 100 μm) and IF staining for macrophage marker F4/80 (scale bar: 50 μm, scale bar of close-up image: 25 μm). **d** Quantification of the diameter of organoids derived from the mice (**c**). **e** IKKβ expression in the colon from normal tissues, uninflamed UC tissues or inflamed UC from the GSE11223 datasets. **f** Representative images of colon from healthy subject and patients with active IBD showing histopathology (scale bar: 200 μm) and the correlation between miR-494-3p and IKKβ (scale bar: 50 μm, scale bar of close-up image: 25 μm). **g** Expression of IKKβ and miR-494-3p in human active IBD samples and normal colon tissues. **h** A schematic figure for the model. Data are presented as mean ± S.D. and analyzed by non-paired two-tailed Student’s *t-*test in the graphs in (**a**–**e**, **g**). *Indicating the significance between WT (+/+) control agomir and miR-494^−/−^ (−/−) control agomir; # indicating the significance between −/− control-agomir and −/− miR-494-3p agomir. ***/^###^*P* ≤ 0.001; **/^##^*P* ≤ 0.01; */^##^*P* ≤ 0.05.

Administration of miRs could be attractive options for the development of therapies for clinical diseases, including IBD, because of their low molecular weight, small size, and amenability to formulation into effective delivery systems^21^. In the present study, we designed a miR-494-3p agomir system in which miR-494-3p agomirs consist of cholesterol-conjugated synthetic RNAs with a 2′-O-methyl linkage and phosphorothioate modification. Chemical modifications have been shown to improve the stability and uptake of the microRNA, in particular providing improved pharmacology^22^. Next, we tested whether administration of this miR-494-3p agomir could elevate miR-494-3p expression in colon and provide in vivo protection from colitis (Supplementary Fig. 7a). We employed qRT-PCR analyses revealed a three- to four-fold increase of miR-494 accumulation in the colon after the treatment with miR-494-3p agomir (Supplementary Fig. 7b). As expected, administration of miR-494-3p agomir in DSS-treated *miR-494*^−/−^ mice delayed disease onset and ameliorated disease severity (Fig. 7a–c). In accordance with these observations, treatment of DSS-treated *miR-494*^−/−^ mice with miR-494-3p agomir resulted in increase in the proliferation of colonic crypt stem cells, and suppressed macrophages infiltration and colonic injury (Fig. 7c, d, Supplementary Fig. 7c). Mechanistically, application of miR-494-3p agomir inhibited the IKKβ/NF-κB signaling in the colon tissues and EDA2R/Wnt/β-catenin signaling in colonic organoids from DSS-treated *miR-494*^−/−^ mice (Supplementary Fig. 7d, e). These data demonstrated that miR-494-3p may serve as a potential target in IBD treatment.

To determine the potential clinical significance of miR-494-3p/IKKβ or EDA-A2/EDA2R in IBD, we analyzed the levels of miR-494-3p, IKKβ, and EDA-A2/EDA2R in experimental colitis and in patients with active IBD. The levels of miR-494-3p in plasma were downregulated in early-stage (day 4) of DSS-induced colitis in our study (Supplementary Fig. 7f). Notably, the analyses of GEO datasets (GSE34874 and GSE22307)^23,24^ showed that the levels of *IKKβ* were elevated in a time-dependent manner in DSS-induced colitis (Supplementary Fig. 7g), which was confirmed by the RNA data from a clinical IBD dataset (GSE11223)^25^ (Fig. 7e). The negative association between miR-494-3p and IKKβ was observed in human active IBD samples (Fig. 7f, g). In addition, our data revealed that the levels of EDA-A2 and EDA2R were enhanced in DSS-induced colitis as well as in active IBD samples (Supplementary Fig. 7h, i).

Therefore, our data demonstrate that the administration of miR-494-3p agomir protects mice from DSS-induced colitis and further show that miR-494-3p negatively correlates with IKKβ and EDA-A2/EDA2R in patients with active IBD.

## Discussion

In the present study, we demonstrated that miR-494-3p deficiency aggravates colitis by integrating the complex cross-talk between differentiated IECs, intestinal stem cells, and macrophages in vivo (Fig. 7h), thus providing an interesting mechanism for the blockage of intestinal crypt cell self-renewal in IBD and a promising target for IBD therapy.

The epithelium in the mucosal surfaces of the colon is self-renewing and is continually replaced every 5–7 days^5^. Thus, the maintenance and differentiation of the intestinal stem cell niches are critical for the IEC homeostasis and IBD recovery, although the underlying mechanisms remain to be fully understood. Our colonic crypt organoid studies showed that organoid proliferation was impaired in crypt cells derived from *miR-494*^−/−^ mice challenged with DSS, where *EDA2R* was the target of miR-494-3p and miR-494-3p depletion activated the interaction between EDA-A2/EDA2R and subsequent signaling. In this context, EDA-A2 was essential for miR-494-3p/EDA2R-induced colonic crypt stem cells destruction, which is consistent with the report that EDA-A2 stabilizes EDA2R and promotes its activation^18^. The homeostatic Wnt/β-catenin pathway is the key regulatory pathway in the intestinal crypts^26^. Recent evidence suggests that progenitors located at the bottom of the crypts accumulate nuclear β-catenin, implying that these cells respond to Wnt stimulation, which has been linked to cell cycle regulators such as c-Myc and cyclin D1^26–28^. Our results revealed that loss of miR-494-3p and EDA2R suppressed the proliferation of crypt progenitor cells otherwise seen upon inhibition of signaling via the β-catenin/c-Myc axis in colonic crypts of DSS-treated mice; notably, this suppression of proliferation was not counteracted by stimulation with exogenous Wnt3a. EDA-A2/EDA2R signaling promotes NF-κB to induce inflammation in diverse physiological and pathological processes, including colorectal cancer^29^. Although our finding that the *IKKβ* 3′-UTR was the target of miR-494-3p in differentiated IECs, we observed no obvious change in NF-κB signaling (Supplementary Fig. 3b) in colonic crypt cells of colitis mice. The underlying mechanism of EDA-A2/EDA2R inhibiting Wnt/β-catenin signaling need to be further studied. Additionally, to identify how miR-494-3p is downregulated in active IBD and experimental colitis, we sought to investigate the potential regulators of miR-494-3p. We also found that the p52/p65 complex suppressed miR-494-3p promoter activation (Supplementary Fig. 9), consistent with another study^30^, thus indicating the existence of negative feedback between NF-κB signaling and miR-494-3p in colitis.

Intestinal macrophages are referred to as a main population of mononuclear phagocytes in the body, critical for innate immunity in IBD^31^. We observed that increased infiltration by F4/80^+^ macrophages, particularly M1 macrophages, in colonic tissues of DSS-treated *miR-494*^−/−^ mice. The cross-talk between IECs and immune cells is known to be dysfunctional in IBD^4,32^. Our data support and expand this idea, indicating that miR-494-3p deletion in differentiated IECs resulted in increased secretion of macrophage-related chemokines (Ccl5/Ccl6 and Cxcl10/12), thereby increasing macrophage recruitment and facilitating M1 polarization in inflamed colon. It appears that miR-494-3p deficiency creates an extremely pro-inflammatory microenvironment that augments the severity of colitis. Under steady condition, intestinal homeostasis is preserved by a population of rapidly dividing stem cells at the bottom of intestinal crypts^5,33^, within which colonic macrophages present a protolerogenic, anti-inflammatory, M2-like phenotype^31,34,35^. However, in the context of pro-inflammatory processes, the responsiveness of intestinal stem cells to macrophage-mediated signaling remains less well understood.

Increasing evidence have suggested that dysbiosis of intestinal microbiota is an important contributor of IBD^1,36^. In the setting of immunoregulatory or mucosal barrier defect, the intestinal microbiota obviously evoke stimulation leading to intestinal inflammation^36^. In our study, miR-494-3p deficiency increased intestinal permeability and dampened crypt cells self-renewal in DSS-induced colitis, suggesting more defective intestinal epithelial barrier in *miR-494*^−/−^ mice. Given that the defective epithelial barrier promotes gut microbiota infiltration, and it may provoke a dimension of immunoregulation and amplify the vicious circle of colitis in *miR-494*^−/−^ mice, which is worthy of further study.

In conclusion, our results demonstrate that miR-494-3p deficiency maintains the cross-talk between IECs (colonic crypt stem cells and differentiated IECs) and macrophages by regulating IKKβ/NF-κB-mediated recruitment of macrophages and EDA-A2/EDA2R-mediated colonic crypt stem cell survival in colitis. Critically, the observations in samples from patients with active IBD samples strongly implied that miR-494-3p plays a protective role in human IBD; thus, analysis and regulation of miR-494-3p expression promise to provide promising diagnostic and therapeutic tools in the management of IBD.

## Methods

### Mice and mouse models

All animals were bred, maintained and used in accordance with the *Guidelines* of the Institutional Animal Care and Use Committee (IACUC) of the Shanghai Institute of Nutrition and Health (SINH) (Shanghai, China). All mice were maintained under specific-pathogen-free conditions in laboratories at 23 ± 3 °C, relative humidity 35% ± 5%, and a 12-h/12-h dark/light cycle with free access to standard diet (Shanghai Laboratory Animal Co. Ltd.; Shanghai, China).

To generate *miR-494*^−/−^ mice, the Sanger MirKO ES cell line miR494 (the whole locus of *miR-494* is knockout in this cell line) was purchased from The Jackson Laboratory (Bar Harbor, ME), and microinjected into C57BL/6 mice at Shanghai Model Organisms (Shanghai, China); resulting male chimeric mice were crossed with female C57BL/6 to generate heterozygous mice, which then were crossed to generate *miR-494*^+/+^ (wild-type, WT) and *miR-494*^−/−^ (knockout, KO) mice. WT and KO mice were kept co-housing until 4 weeks old. The primers sequences for genotyping have been listed in Supplementary Table 2.

For the Dextran Sulfate Sodium (DSS)-induced colitis model, WT and *miR-494*^−/−^ mice (8–12-week-old, male) were provided with free access to 3% DSS (160,110, MW = 36–50 kD; MP Biomedicals, Santa Ana, CA, USA) in the drinking water for 7 days; the first day of DSS access was designated day 0). Mouse body weights and stool appearances were recorded daily. The disease activity index (DAI) was determined as reported previously^37^. Briefly, the DAI involves there parts: body weight change, stool appearance, and rectal bleeding. For the clodronate-mediated macrophage deletion model, mice (8-week-old, male) were injected intraperitoneally (ip) with empty- or clodronate-containing liposomes (200 μl/mice) starting on day-4 (4 days prior to DSS treatment) and again on 0, 2, 4, and 6. To decrease *IKKβ* in colon tissues, *IKKβ* interference adenovirus (Adv-IKKβi) or control adenovirus was administered to mice (8-week-old, male) by rectal instillation on day-1 (1 day prior to DSS treatment). For DSS-induced colitis with miR-494-3p agomir treatment model, control agomir or miR-494-3p agomir (Guangzhou Ribobio Co., Ltd., China) [agomir was dissolved in phosphate-buffered saline (PBS, PH = 7.4)] was administered to mice (8-week-old, male) by intravenous (50 nmol/100 μl/iv) on day 0, 2, 4.

All animal experiments were approved by the Institutional Animal Care and Use Committee at the Shanghai Institutes for Biological Sciences, Chinese Academy of Sciences (Shanghai, China).

### Isolation of lamina propria lymphocytes (LPL) and of splenic immune cells

For isolation of LPL, the whole colons from 8 to 10-week-old male mice were removed, cut into fragments of about 1 cm in length, and rinsed with HBSS buffer. The rinsed colon fragments then were gently shaken (250 rpm) for 30 min at 37 °C in HBSS buffer supplemented with 30 mM EDTA (sigma, Darmstadt, Germany), 1 mM DTT (sigma), and 5% (vol/vol) FBS. After sedimentation by standing, the supernatant was discarded and the remaining colon tissues were further cut into smaller pieces and incubated with digestive solution [RPMI-1640 medium (Gibco) supplemented with 200 U/mL collagenase VIII (sigma), 150 μg\mL DNAase I (sigma), 5% (vol/vol) FBS, and 1% (vol/vol) penicillin and streptomycin] for 1 h at 37 °C. After digestion, the supernatant was centrifuged at 450 × *g* for 5 min and Percoll (40%/80%; GE Healthcare) was used to isolate LPLs. The LPLs then were subjected to flow cytometry or culture in RPMI-1640 supplemented with 10% (vol/vol) FBS and 1% (vol/vol) penicillin and streptomycin for 48 h.

For isolation of immune cells from the spleen, spleen was crushed and passed through a 40-μm filter to obtain a single-cell suspension. After centrifugation at 300 × *g* for 5 min, cells were resuspended in red blood cell lysing buffer for 5 min and then again passed through a 40-μm filter. The resulting suspension was subjected to another round of centrifugation at 300 × *g* for 5 min and the resulting supernatant was discarded. The remaining cells were washed 2 times with PBS (pH = 7.4) and then subjected to flow cytometry.

### Human cohort

Colon tissues and sera were collected from IBD patients who had been pathologically confirmed to have active UC (A-UC) or CD (A-CD). All of patients were recruited from the Shanghai Tenth People’s Hospital of Tongji University (Shanghai, China). The diagnosis of active IBD was performed as previously described^11^. Colon tissues were taken at the time of endoscopy and uninflamed tissues from the respective patient were used as controls. Sera from healthy volunteers and active IBD patients were collected in the course of blood collection for clinical examination. The characteristics of IBD patients and healthy donors are listed in Supplementary Table 1. The use of human specimens was approved by the Ethics Review Board of the Shanghai Tenth People’s Hospital (Tongji University). Before the study, we obtained written informed consent from all participants.

### Colon organoid culture

Following euthanasia of 8 to 10-week-old male mice, the entire colon was removed, everted, and rinsed 5 times with cold PBS (supplemented with 2% (vol/vol) penicillin and streptomycin). The rinsed colon was immersed into chilled (0–4 °C) cell recovery solution buffer (354253, Corning, NY, USA) for 30–40 min; the resulting cell suspension was filtered through 70-μm strainers and rinsed with basic cell culture medium (DMEM/F12 (Gibco^TM^, Waltham, MA) supplemented with 1% (vol/vol) penicillin and streptomycin (Gibco^TM^), 1% glutamax (Gibco Gibco^TM^), and 1% HEPES (Gibco^TM^)) until the supernatant remained clear after centrifugation at 200 × *g* for 2–3 min. The rinsed colonic crypts were resuspended in Matrigel (354230, BD) and seeded in pre-warmed 24-well culture plates; the plates were incubated in cell culture incubator for 10 min, and colon organoid growth medium (consisting of a 1:1 mixture of DMEM/F12:L-Wnt 3 A supernatant supplemented with 20% (vol/vol) FBS (BI), 1% (vol/vol) penicillin and streptomycin, 500 ng/ml R-Spondin (3474-RS, R&D), 50 ng/ml EGF (50482-M01H, Sino Biological Co., Ltd., China), 100 ng/ml Noggin (250-38-20, Peprotech), 10 μM Y-27632 (1254, Tocris)) was distributed to the culture plate at 500 μL/well (the nominal day 0). To characterize the surviving rate of those organoids, the same number crypts (around 100 crypts/50 μl per well) were plated at day 0, which were cultured in the organoids culture from WT mice or *miR-494*^−/−^ mice at day 0, was equal. After culturing for the indicated times, organoids were subjected to western blot or qRT-PCR analysis. For EDA-A2 treatment assay, 5 μg/mL EDA-A2 (922-ED, R&D) was added to the growth medium at day 2; for miR-494-3p agomir interference studies, miR-494-3p agomir or control agomir was added to the growth medium at day 1; for the experiment examining the effect of conditional meidum from LPL or BMDM, the culture medium was replaced with a 1:1 mixture of colon organoid growth medium and the supernatant from conditioned growth medium from LPL or BMDM cells at day 2.

The L-Wnt 3 A cell line was purchased from Shanghai Fuxiang Biotechnology Co., Ltd. and cultured in growth medium (DMEM supplemented with 10% FBS and 1% (vol/vol) penicillin and streptomycin) to 80–90% cell confluence; the resulting conditioned medium was collected every three days for a total of three times and filtered through 0.22-μm filters.

### Isolation of intestinal epithelial cells (IECs)

For each of the indicated male mice (3 to 4-week-old), the whole colon was removed and cut into 1-mm^3^ pieces, which were subjected to 4–5 rounds of rinsing with cold PBS (pH = 7.4) containing 2% (vol/vol) penicillin and streptomycin. The tissues then were incubated in a digestive solution [DMEM supplemented with 5% (vol/vol) FBS, 0.8 mg/mL collagenase XI (sigma), 4 μg/mL dispase (BD Biosciences, San Jose, CA), and 2% (vol/vol) penicillin and streptomycin] for 3–4 h at 37 °C. The digested supernatant was centrifuged at 300 × *g* for 5 min and rinsed 5 times with cold wash buffer [DMEM supplemented with 2.5% (g/vol) D-sorbitol (sigma), 2% (vol/vol) penicillin and streptomycin, and 20% (vol/vol) FBS]. The resulting isolated IECs were seeded on Matrigel-coated plates and cultured in growth medium [DMEM/F12 supplemented with 1% (vol/vol) insulin mix, 2.5% (vol/vol) FBS, and 2% (vol/vol) penicillin and streptomycin].

For the LPS treatment assay, IECs were cultured for 4–5 days and then incubated with 1 μg/mL LPS for indicated time. For the miR-494-3p overexpression assay, IECs were cultured for 4–5 days and then transfected with the miR-494-3p mimic or control mimic; after 24 h, the transfected cells were subjected to LPS treatment for the indicated time.

### Bone marrow-derived macrophage (BMDM) culture and chemotaxis assay

Briefly, BMDMs from femoral bones of 8-wk-old male mice were cultured in BMDM growth medium [RPMI-1640:L929 supernatant: FBS = 5:3:2 (vol/vol) supplemented with 1% (vol/vol) penicillin and streptomycin] for 1 week, and the cells then were stimulated with indicated cytokines (100 ng/mL LPS (sigma), 2 ng/ml IL-6 (R&D), 10 ng/ml IL-1β (R&D), or 10 ng/ml IL-4 (R&D)) or conditioned medium from IECs for the indicated time.

The chemotaxis assay was performed in 24-well inserts with 8-μm pores (Corning, Inc., Corning, NY, USA). BMDMs or RAW264.7 cells (4 × 10^4^/well in 200 μL blank medium supplemented with 0.2% (g/ml) BSA) were seeded in the upper side of each insert and 600 μL of conditioned medium from IECs was added to the bottom well. Following incubation for 24 h, the migrating cells were fixed with 4% (g/ml) polyoxymethylene (PFA, sigma) for 30 min and stained with 0.1% (g/ml) crystal violet solution for another 20 min. Remaining cells on the upper side of insert were removed. Following drying at room temperature, images were captured using an Olympus IX51 microscope and the numbers of cells were counted using ImageJ software.

### Cytokine and chemokine assay from serum or IEC supernatant

Sera from mice provided with free access to 3% DSS in drinking water for 7 days was assessed for the levels of cytokines. Conditioned medium from IECs subjected to 48 h of the indicated treatment was collected and centrifuged at 3000 × *g* for 15 min; the resulting supernatant was assessed for the levels of cytokines and chemokines. All levels of cytokines and chemokines were determined using commercially available ELISA kits (R&D) according to the manufacturer’s protocols.

### Histopathology, immunohistochemistry (IHC), immunofluorescence (IF), and TUNEL assay

4% PFA-fixed and paraffin-embedded colon tissues were sectioned at 5-μm thicknesses and transferred to glass slides. These sections were subjected to hematoxylin-eosin (H&E), IHC, IF, or TUNEL assay according to standard protocols and then examined by light microscopy. For H&E staining, stained sections were scored for colonic histology as reported previously^38^ by evaluators blinded to the sample identities. Briefly, histology score is a combination of tissue damage (0, no mucosal damage; 1, mucosal lesions; 2, surface mucosal erosion or focal ulceration; 3, extensive mucosal damage and extension into deeper structures of the bowel wall), inflammatory cells infiltration (0, no infiltration; 1, slightly dispersed cells infiltration; 2, severely large area of cell infiltration causing loss of tissue structure), and crypt injury (0, none; 1, basal 1/3 damaged; 2, basal 2/3 damaged; 3, loss of surface epithelium; 4, loss of epithelium and crypts). For IHC staining, colon tissues were incubated with anti-Ki67 antibody (NBP1-40684, Novus); for IF staining, colon tissues were incubated with primary antibodies against the following markers: F4/80 (30325, Cell Signaling Technology; Boston, MA, USA) and Ki67 (ab15580, Abcam, Cambridge, UK) and then with secondary antibodies: goat anti-rabbit IgG highly cross-adsorbed secondary antibody, Alexa Fluor Plus 488 (A32731, ThermoFisher Scientific, Waltham, MA) or 568 (A-11011, ThermoFisher Scientific); for TUNEL assay, the assay was performed using the TUNEL FITC Apoptosis Detection Kit (A111-01, Vazyme Biotech Co.,Ltd, China) according to the manufacturer’s protocols. ImageJ was used to quantify the positive signaling in our study. Once we got the average staining intensity value from ImageJ, all values were normalized by the minimal value. The relative intensity is presented in figures.

### Flow cytometry

LP cells from colon or splenic immune cells were sorted using a FACS flow cytometer (BD Biosciences, San Jose, CA) and analyzed using FlowJo_V10 (Treestar, Ashlan, OR). The gating strategy has been shown in supplementary Figure 8. For macrophage and neutrophil analyses, cells were incubated with the following antibodies: FITC-conjugated anti-CD11b (11-0112-82, eBioscience, San Diego, CA), APC-conjugated anti-CD11c (17-0114-81, eBioscience), PE-cyanine7-conjugated anti-F4/80 (25-4801-82, eBioscience), PE-conjugated anti-CD206 (12-2061-80, eBioscience), and PE-conjugated anti-Ly6G (12-5931-81, eBioscience); for T cell analyses, cells were incubated with the following antibodies: APC-conjugated anti-CD3 (17-0032-82, eBioscience), FITC-conjugated anti-CD4 (11-0042-81, eBioscience), and PE-conjugated anti-CD8 (12-0081-81, eBioscience); for B cell and NK cell analyses, cells were incubated with the following antibodies: FITC-conjugated anti-CD19 (11-0193-81, eBioscience), and PE-conjugated anti-NK1.1 (12-5941-81, eBioscience).

### Western blot analysis

Western blot was performed as described previously^39^. Blots were probed with antibodies against IKKβ, p-p65, p65, Hsp90, β-catenin (Cell Signaling Technology; Boston, MA, USA); antibody against c-Myc (Santa Cruz Biotechnology (Santa Cruz, CA, USA); antibodies against β-actin (Yeasen Biotechnology Co., Ltd.; Shanghai, China); and antibody against EDA2R (Abcam).

### Real time-PCR and quantitative reverse transcription-PCR (qRT-PCR)

Cells or tissues from the indicated mice were extracted using Trizol reagent (Takara, Dalian, China) and RNA was isolated according to the manufacturer information. U6 RNA, cel-miR-39, and the transcript encoding *β-actin* were used to normalize gene expression levels. The primers sequences are listed in Supplementary Tables 3 and 4.

### Bioinformatics

miR-494-3p targets were predicted by using the online websites miRbase (http://www.mirbase.org) Targetscan (http://www.targetscan.org), and miRDB (http://www.miRdb.org). Colitis datasets were obtained from GEO datasets.

### Statistics and reproducibility

Experimental data were analyzed by unpaired, two-tailed Student’s *t*-tests and are presented as the mean ± S.D.; analyses were conducted using Prism 6.01 software (GraphPad; San Diego, CA). *P* values of less than 0.05 were considered significant. Sample sizes are indicated in figure legends and methods and all experiments have been repeated at least three times.

### Reporting summary

Further information on experimental design is available in the Nature Research Reporting Summary linked to this paper.

## Supplementary information

Supplementary Information

Description of Additional Supplementary Files

Supplementary Data 1

Reporting Summary

## Data Availability

All of the sequencing data that support the findings of this study have been deposited in the NCBI under accession codes GSE137889, GSE137890, and GSE137892. Previously published deep-seq data that were re-analyzed here are available under accession codes GSE34874, GSE68306, GSE22307, and GSE11223. Source data underlying plots shown in figures are provided in Supplementary Data 1. Full blots are shown in Supplementary Information. All other data supporting the findings of this study are available from the corresponding author on reasonable request.
