# Supplementary material for: Macrophage-derived EDA-A2 inhibits intestinal stem cells by targeting miR-494/EDA2R/β-catenin signaling in mice
Source: Commun Biol. 2021 Feb 16;4:213. doi: 10.1038/s42003-021-01730-0 (PMC7887198; doi:10.1038/s42003-021-01730-0)
Supplement: Supplementary file 1 — Supplementary Information [file 42003_2021_1730_MOESM1_ESM.pdf]

Supplementary Information

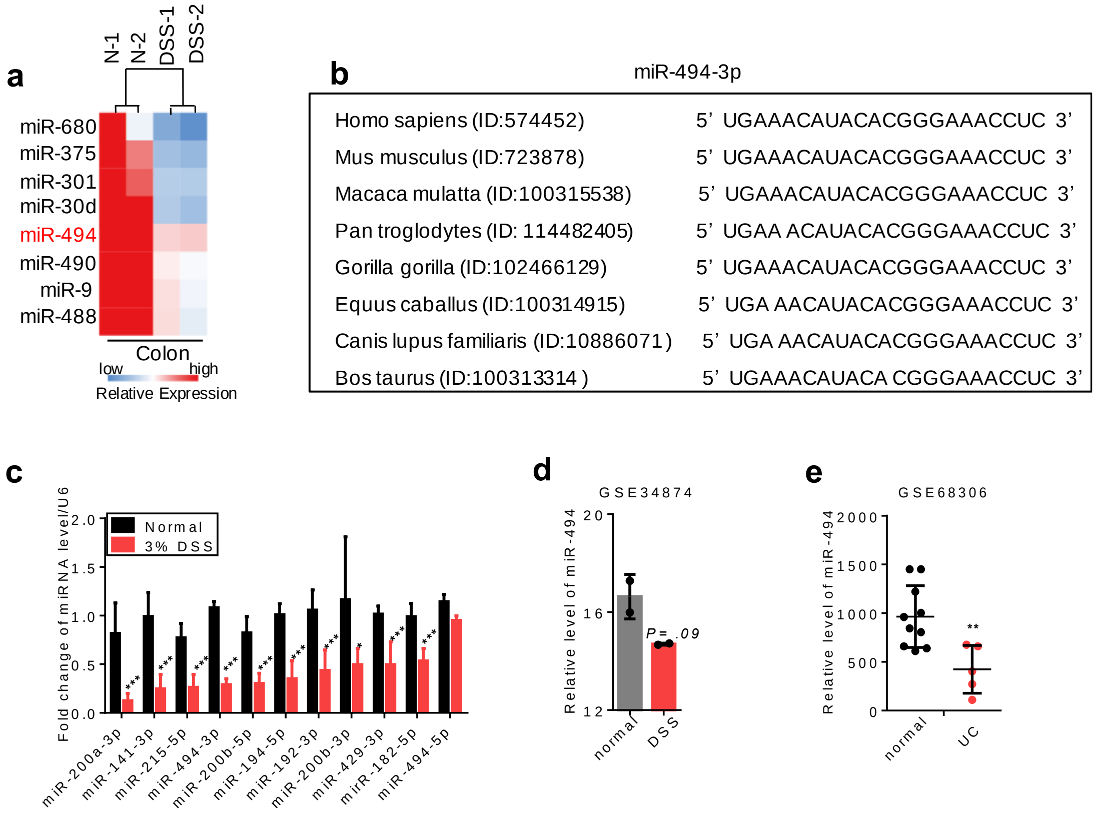

Supplementary Figure 1 miR-494-3p deficiency may be related to colitis. a microRNA expression profile in the colon tissues from normal mice (N-1, N-2) or DSS-treated mice (DSS-1, DSS-2) from the GSE34874 dataset[1]. **b** Sequences of miR-494-3p in the indicated species. **c** C57BL/6 mice (male, 8-week-old) were divided into two groups and provided with drinking water neat (Normal) or drinking water with 3% DSS (3% DSS) for 7 days. At day 7, colonic crypts were isolated and RNAs were subjected to miRNAome analysis. microRNAs whose levels were decreased significantly in microarray were assessed by qRT-PCR (normalized to that of U6). **d** miR-494 expression in the colon tissues from normal mice and DSS-treated mice from the GSE34874 dataset. **e** miR-494 levels in colon tissues from healthy subjects or patients with ulcerative colitis (UC) from the GSE68306 dataset[2]. Data are presented as mean±S.D. and analyzed by non-paired two-tailed Student's *t*-test in panels (**c-e**). ***$P \leq 0.001$, **$P \leq 0.01$; NS, not significant.

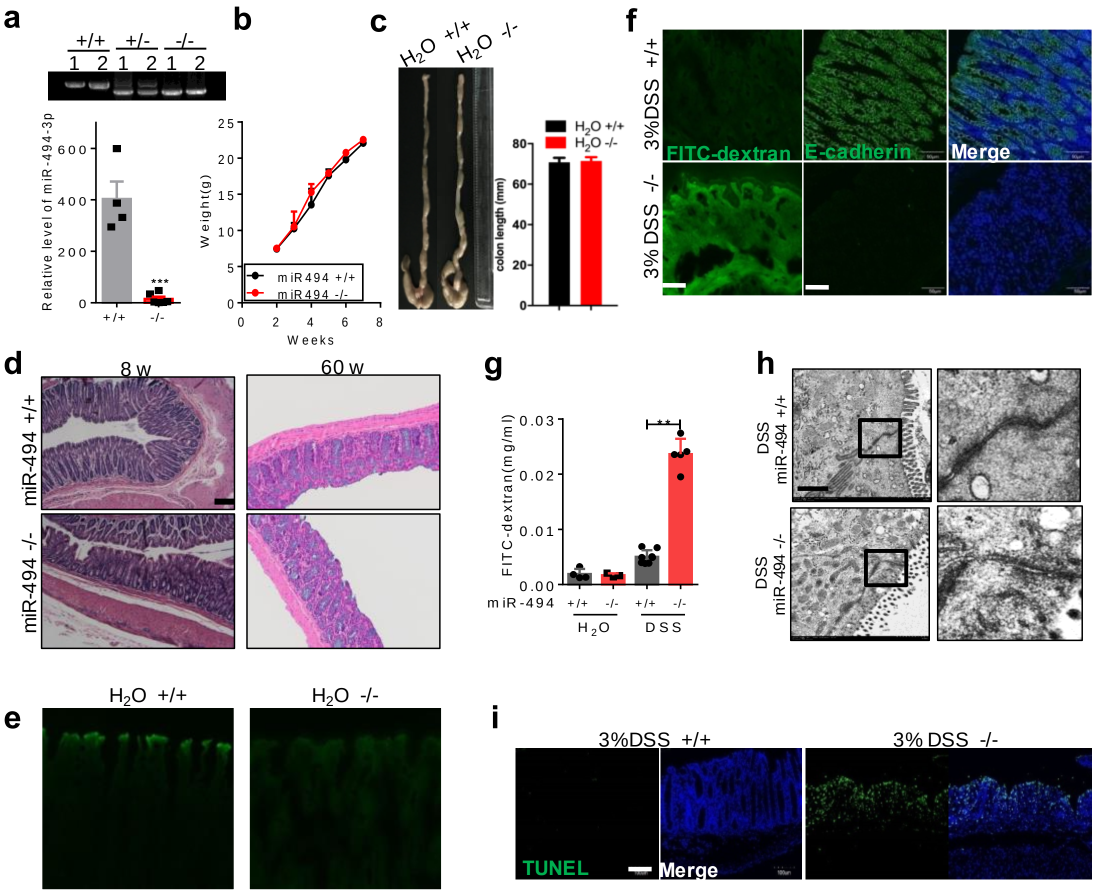

Supplementary Figure 2 *miR-494*$^{-/-}$ mice exhibit abnormalities in crypt stem cell identity in DSS-induced colitis. **a** Genotyping by genomic PCR analysis. +/+, wide type (WT); +/-, heterozygous for miR-494 knockout allele; -/-, homozygous for miR-494 knockout allele. miR-494-3p levels in colon tissues from *miR-494*$^{+/+}$ and *miR-494*$^{-/-}$ mice were measured by qRT-PCR. The sequences of primers are listed in Supplementary Table 2. **b-e** Male mice were provided with free access to normal drinking water. Body weight (**b**); representative images and quantitative plot of colon length (**c**); typical hematoxylin and eosin (H&E) staining images of colon tissues [left, 8 weeks (8w); right, 60 weeks (60w); scale bar: 200μm) (**d**); intensity of FITC-dextran accumulation in colon mucosa (**e**, scale bar: 50μm) from male mice (8-week-old). **f** Representative images of fluorescence from FITC-dextran accumulation (left column) and immunofluorescent (IF) staining for epithelial marker E-cadherin (middle column) in surface of colon mucosa of mice provided with free access to drinking water with 3% DSS for 7 days. Right column provides merged images from preceding columns (scale bar: 100μm). **g** Intensity of FITC-dextran in plasma was measured in mice provided with free access to normal drinking water or drinking water with 3% DSS for 7 days. **h** Representative Transmission Electron Microscope (TEM) images of tight junction in colon epithelial cells in mice provided with free access to drinking water with 3% DSS for 7 days. (scale bar=1μm). **i** TUNEL assay for colon tissue of mice provided with free access to drinking water with 3% DSS for 7 days (scale bar: 50μm). Data are presented as mean±S.D. and analyzed by two-tailed non-paired Student's *t*-test in the graph in panels (**a**, **g**). ***$P \leq 0.001$.

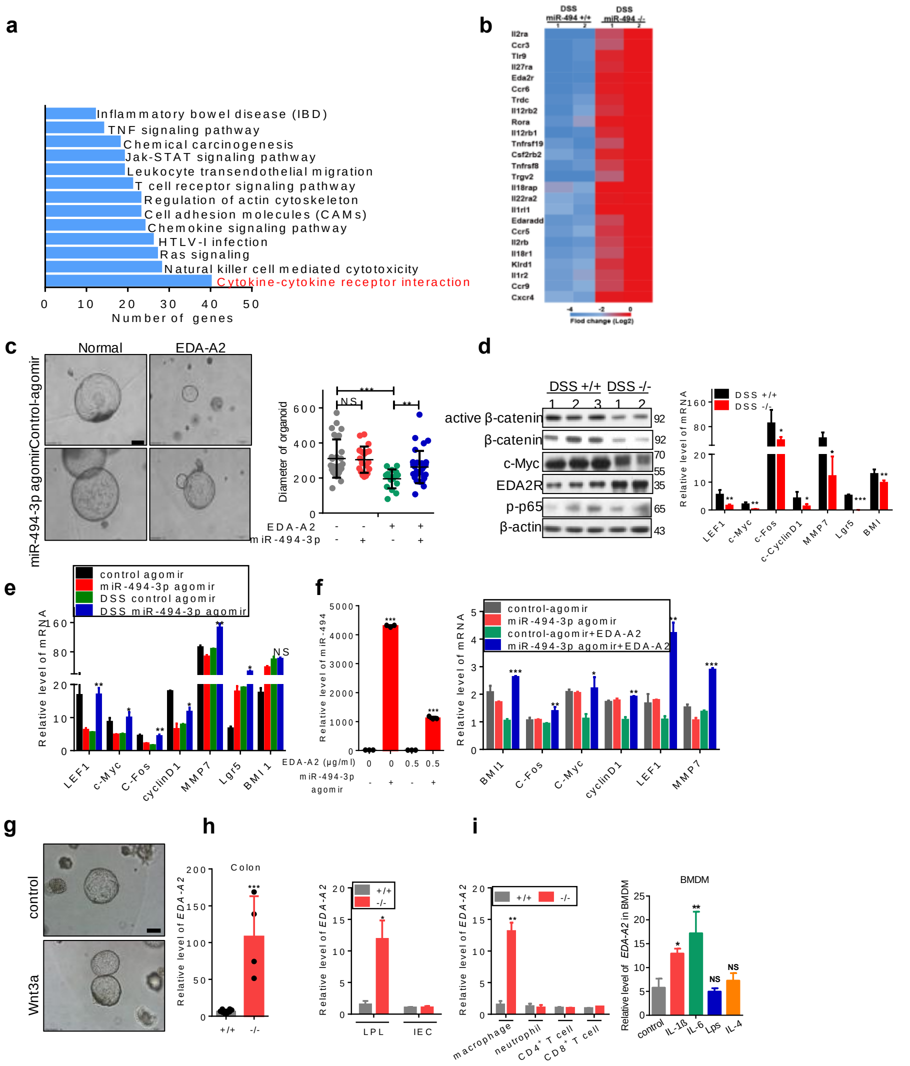

Supplementary Figure 3 Identification of a new cytokine (EDA-A2) and its receptor (EDA2R) that attenuates crypt stem cells proliferation in DSS-induced colitis. a-b Colonic crypts were extracted from mice provided with free access to drinking water supplemented with 3% DSS for 7 days; RNA was prepared, and RNA-seq was then performed. **a** Statistical analysis of pathway enrichment for crypts from DSS-treated *miR-494*$^{-/-}$ mice VS crypts from DSS-treated WT (+/+) mice. **b** Heatmap showing relative expression intensity of > 4 folds differentially expressed cytokine receptors in the crypts between miR-494-/- mice and WT (+/+) mice. **c** Representative images and quantification of the size of colonic organoids. Organoids incubated with miR-494-3p agomir or control agomir were treated with or without EDA-A2 for 8 days (scale bar: 100µm). **d** Western blot and qRT-PCR analyses were used to determine the signaling of colonic crypts from *miR-494*$^{-/-}$ mice and WT (+/+) mice treated with 3% DSS in the drinking water for 7 days. **e** The levels of selected mRNAs (the indicated markers of proliferation and stemness) in colon organoids were determined by qRT-PCR. The organoids were derived from colon crypt cells that were extracted from mice provided with free access to drinking water neat or supplemented with 3% DSS for 7 days; during the organoid culture, the organoids were incubated with control agomir or miR-494-3p agomir for 4 days. **f** The levels of selected mRNAs (the indicated markers of proliferation and stemness) in colon organoids were determined by qRT-PCR. The organoids were derived from colon crypt cells that were extracted from mice provided with free access; during the

organoid culture, the organoids were incubated with EDA-A2 for 7 days and control agomir or miR-494-3p agomir for first 4 days. **g** Representative images of organoids derived from DSS-treated *miR-494*$^{-/-}$ mice; the organoids were cultured in the presence or absence of Wnt3a (scale bar: 100μm). **h** The levels of *EDA-A2* in colon tissues, LPL, and IECs were determined by qRT-PCR. Colon tissues, LPL and IEC were obtained from mice provided with free access to 3% DSS in drinking water for 7 days. **i** The levels of EDA-A2 in different immune cells from LPL (left) or BMDM (right). Immune cells were sorted from LPL of mice treated with 3% DSS in drinking water for 7 days; BMDMs were incubated with indicated cytokines (IL-1β, IL-6, LPS, and IL-4) for 12 hours. All qRT-PCR values were normalized against the expression of the transcript encoding β-actin. Data are presented as mean±S.D. and analyzed by non-paired two-tailed Student's *t*-test in panels (**c**-**e**, **h**, **i**). ***$P \leq 0.001$, **$P \leq 0.01$, *$P \leq 0.05$; NS, not significant.

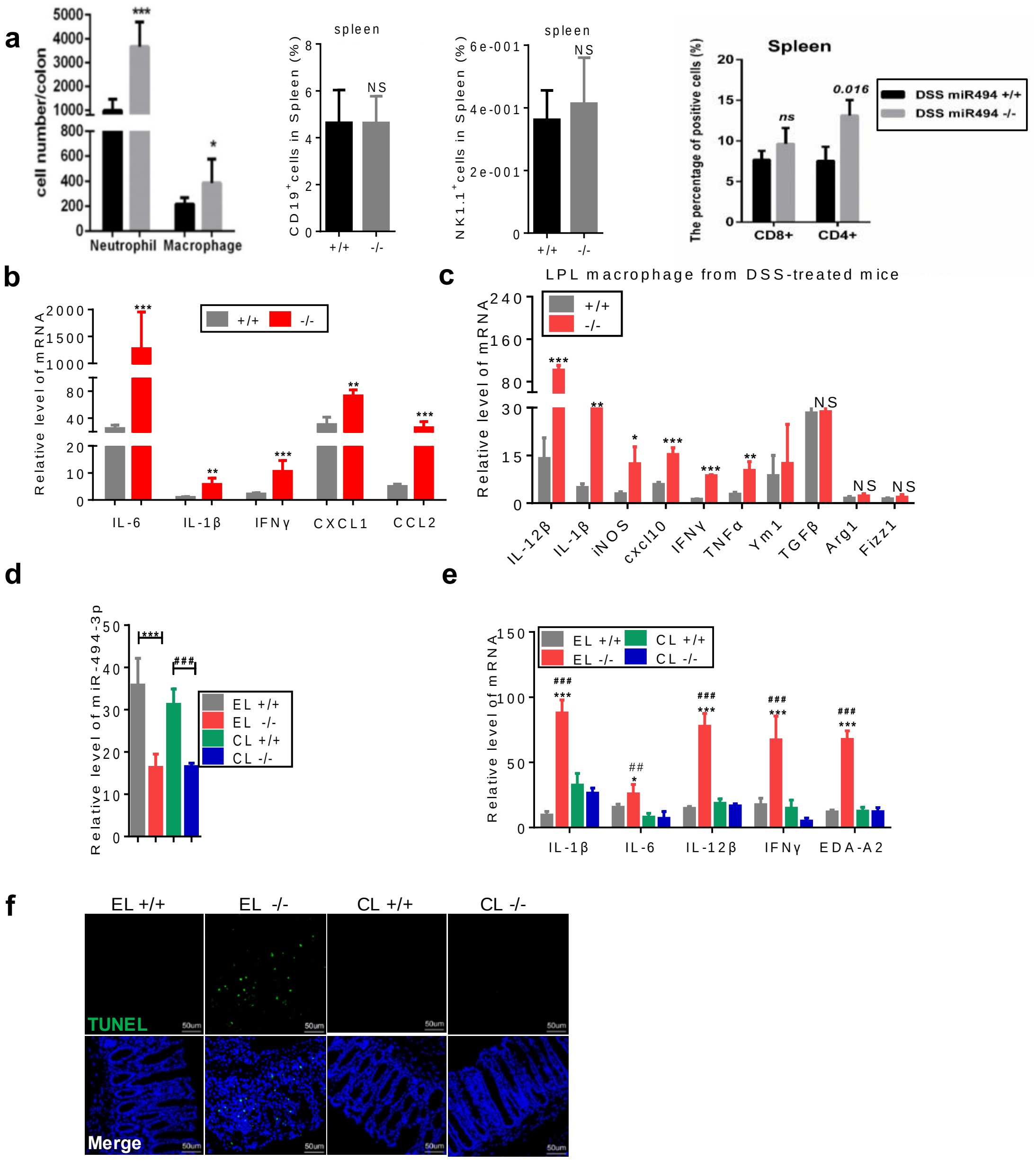

Supplementary Figure 4 LP macrophages contribute to the increased inflammation in colitis of in DSS-treated *miR-494*$^{-/-}$ mice. a Subpopulations of colonic LP neutrophils (gated for CD11b^{+}Ly6G^{hi}) and macrophages (gated for CD11b^{+}F4/80^{+}); and subpopulations of splenic CD4 cells (gated for CD3^{+}CD4^{+}), CD8 cells (gated for CD3^{+}CD8^{+}), B cells (gated for CD19^{+}NK1.1^{-}), and NK1.1 cells (gated for CD19^{-}NK1.1^{+}) were analyzed from *miR-494* KO (-/-) and wild-type (+/+) mice on day 7 after DSS treatment. **b-c** The mRNA levels of each of the indicated cytokines and chemokines was determined by qRT-PCR analysis in colon tissues (**b**) and in LPL macrophages (**c**) from mice provided with free access to drinking water containing 3% DSS treatment for 7 days. **d-f** Wild-type mice (+/+) and *miR-494*$^{-/-}$ mice (-/-) were provided with free access to 3% DSS in drinking water; and empty liposomes (EL) or clodronate liposomes (CL) were administered by ip injection on days -4, 0, 2, 4, and 6. **d** miR-494-3p levels in colon tissues were measured by qRT-PCR. **e** The mRNA levels of each of the cytokines and chemokines was determined by qRT-PCR analysis in colon tissues. **f** Representative images of colon tissues stained

by the TUNEL assay (scale bar: 50μm). All qRT-PCR values were normalized against the expression of the transcript encoding β-actin. Data are presented as mean±S.D. and analyzed by two-tailed non-paired Student's *t*-test in the graphs in panels (**a**-**e**). ***$P \leq 0.001$; **$P \leq 0.01$; *$P \leq 0.05$; NS, not significant.

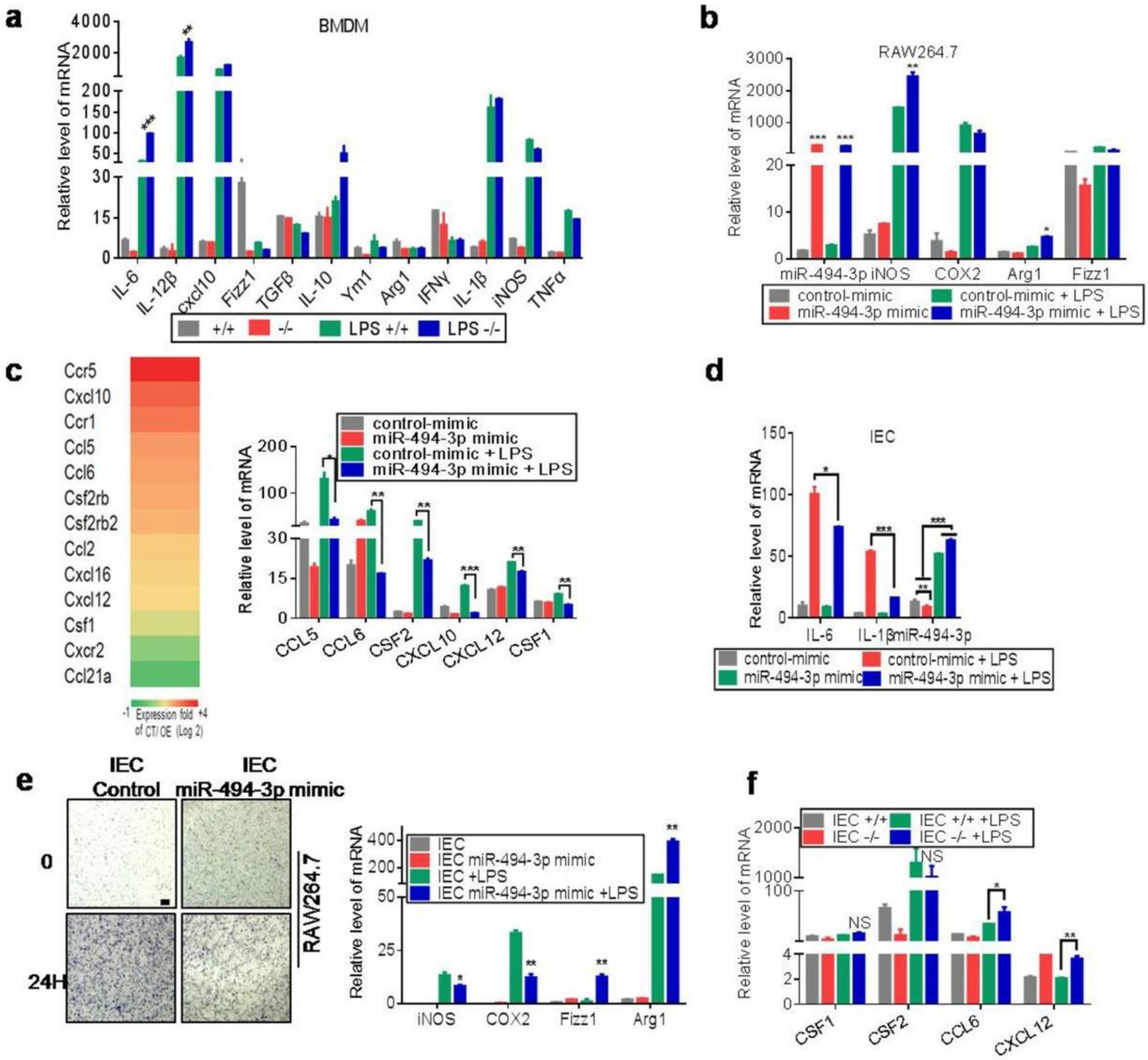

Supplementary Figure 5 Depletion of miR-494-3p in intestinal epithelial cells (IECs) promotes macrophage recruitment and M1 polarization in colitis. a The mRNA levels of M1 and M2 markers in bone marrow derived macrophage (BMDM) were measured by qRT-PCR analysis. BMDM was isolated from *miR-494*$^{-/-}$ mice or WT (+/+) mice, treated with or without LPS. **b** The mRNA levels of M1 and M2 markers in RAW264.7 cells were measured by qRT-PCR analysis. The cells were transfected with control mimic or miR-494-3p mimic and then (after 24 hours later) treated with or without LPS. **c** Heat map showing relative expression intensity of different chemokines between LPS-treated IECs transfected with control mimic (CT) or miR-494-3p mimic (OE). The mRNA levels of macrophage-related chemokines were measured by qRT-PCR in IECs subjected to the indicated treatment. **d** The mRNA levels of IL-1β, IL-6 and miR-494-3p were measured by qRT-PCR analysis in IECs subjected to 24 hours treatment with or without LPS. **e** Representative images of migrating RAW264.7 cells following incubation with conditioned medium from IECs that had been transfected with control mimic or miR-494-3p mimic. Scale bar: 200μm. The mRNA levels of M1 and M2 markers in RAW264.7 cells were measured by qRT-PCR analysis following incubation of cells in conditioned medium from IECs

derived from *miR-494*$^{-/-}$ mice or WT mice treated with or without LPS. **f** The mRNA levels of macrophage-related chemokines were measured by qRT-PCR in IECs from *miR-494*$^{-/-}$ mice and WT mice that had been treated with LPS treatment for 24 hours. All qRT-PCR values were normalized against the expression of the transcript encoding *β-actin*; the value of miR-494-3p was normalized against the expression of U6. Data are presented as mean±S.D. and analyzed by non-paired two-tailed Student's *t*-test in the graphs in panels (**a-f**). $***P \leq 0.001$; $**P \leq 0.01$; $*P \leq 0.05$.

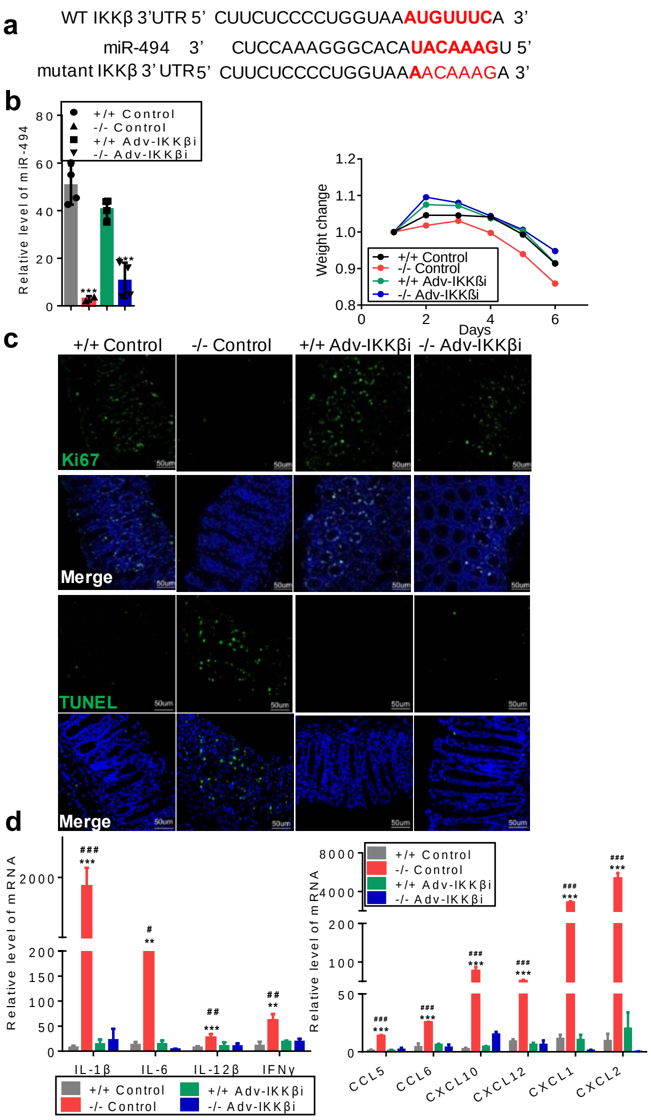

Supplementary Figure 6 miR-494-3p in IECs directly targets *IKKβ* to integrate IECs and macrophages signaling in colitis mice. a Sequences of mouse miR-494-3p and *IKKβ* 3' UTRs. **b-d** *IKKβ* interference adenovirus (Adv-IKKβi) or control adenovirus was administered to *miR-494*$^{-/-}$ and WT (+/+) mice by coloclysis and the mice then were provided with free access to 3% DSS in drinking water for 7 days. **b** miR-494-3p levels in colon tissues were determined by qRT-PCR; expression was normalized to that of U6. Additionally, body weight changes were tracked in these mice during the study. **c** Colon tissues were subjected to the IF staining for the proliferation marker Ki67 and TUNEL assay (scale bar: 50μm). **d** The mRNA level of each

indicated cytokines or chemokines in colon tissues was measured by qRT-PCR. Except as indicated, qRT-PCR values were normalized against the expression of the transcript encoding β-actin. Data are presented as mean±S.D. and analyzed by two-tailed non-paired Student's *t*-test in the graphs in panels (**b**, **d**). *indicating the significance between WT (+/+) mice and *miR-494*$^{-/-}$ (-/-) mice; # indicating the significance between KO mice with Adv-control and KO mice with Adv-IKKβi. ***/### P ≤ 0.001, **/## P ≤ 0.01.

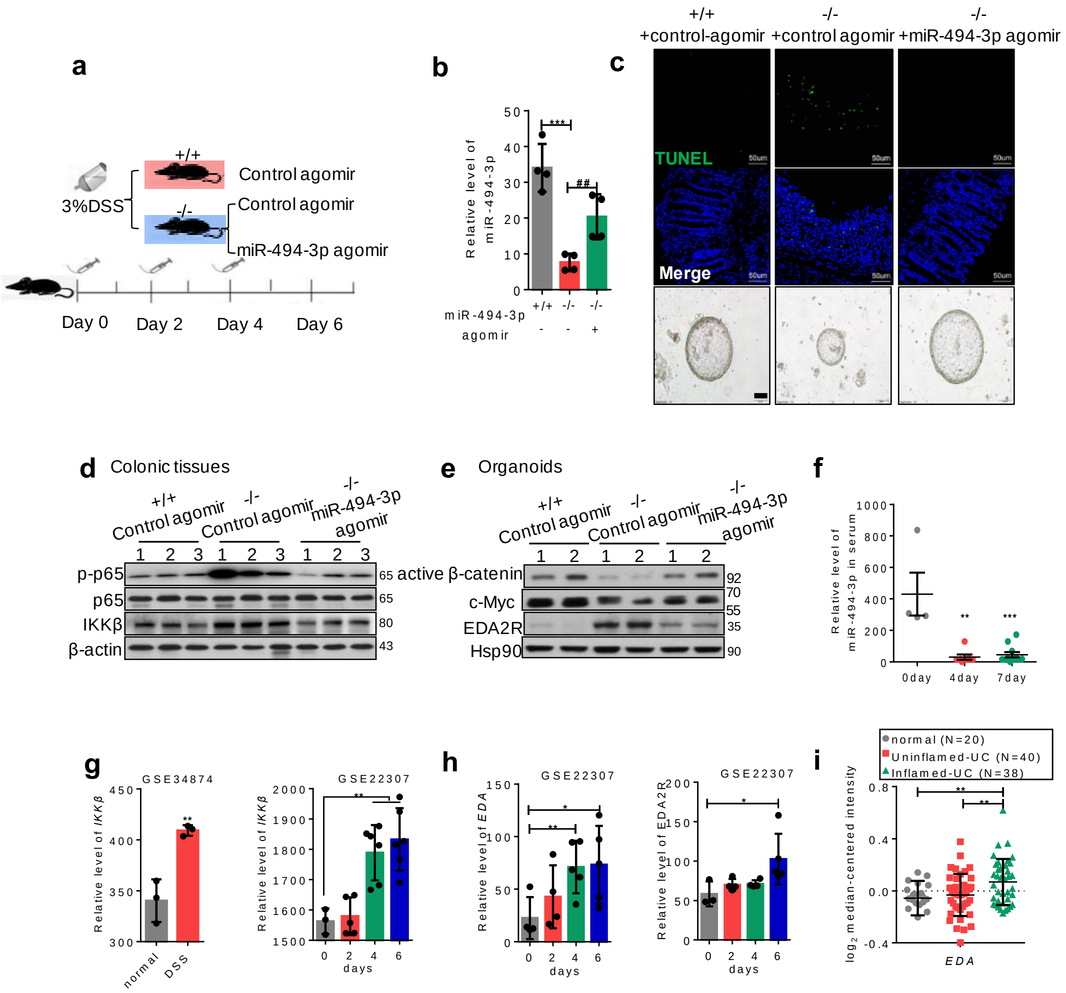

Supplementary Figure 7 Administration of miR-494-3p agomir protects mice from colitis. a-d *miR-494*$^{-/-}$mice and WT mice (+/+) (male, 8-week-old) were provided with free access to 3% DSS in drinking water for 7 days. miR-494-3p agomir or control agomir was administrated by injection at day 0, 2, 4 (where day 0 is in the initiation of DSS treatment). **a** A schematic timeline of the miR-494-3p agomir treatment assay. **b** miR-494-3p levels in colon tissues from mice at day 7 were determined by qRT-PCR (normalized by U6). **c** The top two rows: TUNEL assay for colon tissues from mice at day 7 (scale bar=50μm); the last row: representative images (scale bar= 100μm) of colon organoids derived from crypt cells isolated from mice subjected to the above treatment and then cultured *in vitro* for 6 days. **d** Western blot of colon tissues for NF-κB signaling, including p-p65, p65, and IKKβ, as well as for the housekeeping protein β-actin (as a loading control). **e** Western blot of organoids for EDA2R/β-catenin/c-Myc signaling as well as for the

housekeeping protein Hsp90 (as a loading control). **f** miR-494-3p levels in colon tissues from WT mice provided with 4 or 7 days of free access to 3% DSS in drinking water. miR-494-3p levels were determined by qRT-PCR and expression was normalized to that of U6. **g** IKKβ expression in colon tissues from normal-treated mice or DSS-treated mice from the GSE34874 and GSE22307 datasets. **h** EDA and EDA2R expression in colon tissues from normal or DSS-treated mice from the GSE22307 dataset[3]. **i** *EDA* expression in the colon from normal tissue, un-inflamed UC tissue, or inflamed UC from the GSE11223 dataset[4]. Data are presented as mean±S.D. and analyzed by non-paired two-tailed Student's *t*-test in the graphs in panels (**b**, **f-i**). ***$P \le 0.001$, **$P \le 0.01$, *$P \le 0.05$; NS, not significant.

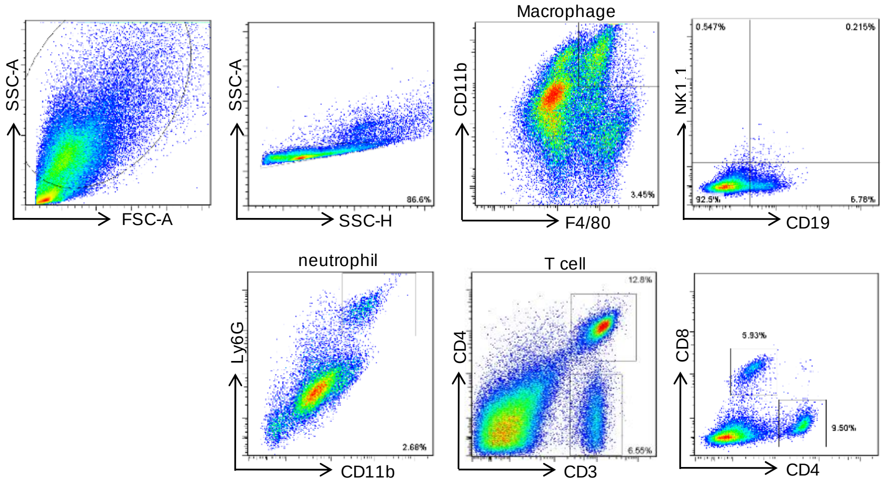

Supplementary Figure 8 The gating strategy of flow cytometry. Subpopulations of colonic LP neutrophils (gated for $CD11b^+Ly6G^{hi}$) and macrophages (gated for $CD11b^+F4/80^+$); and subpopulations of splenic CD4 cells (gated for $CD3^+CD4^+$), CD8 cells (gated for $CD3^+CD8^+$), B cells (gated for $CD19^+NK1.1^-$), and NK1.1 cells (gated for $CD19^-NK1.1^+$) were analyzed from *miR-494* KO (-/-) and wild-type (+/+) mice on day 7 after DSS treatment.

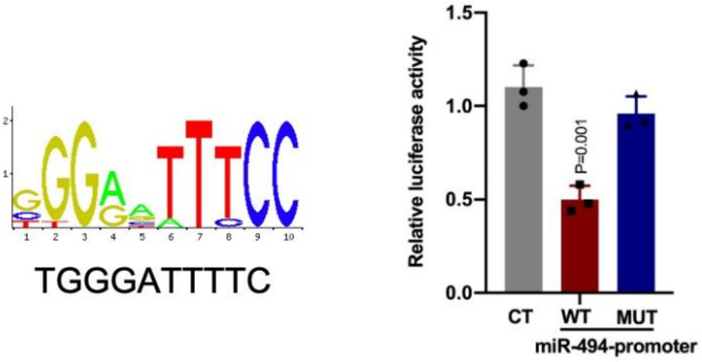

Supplementary Figure 9 p52/p65 comple x suppressed miR-494-3p promoter activation. Sequences of miR-494 promoter which are bound with p65/p52. Luciferase activity of the reporter vector containing the WT or mutant (MUT) miR-494 promoter was determined after co-transfection with control or pCMV-p52/p65.

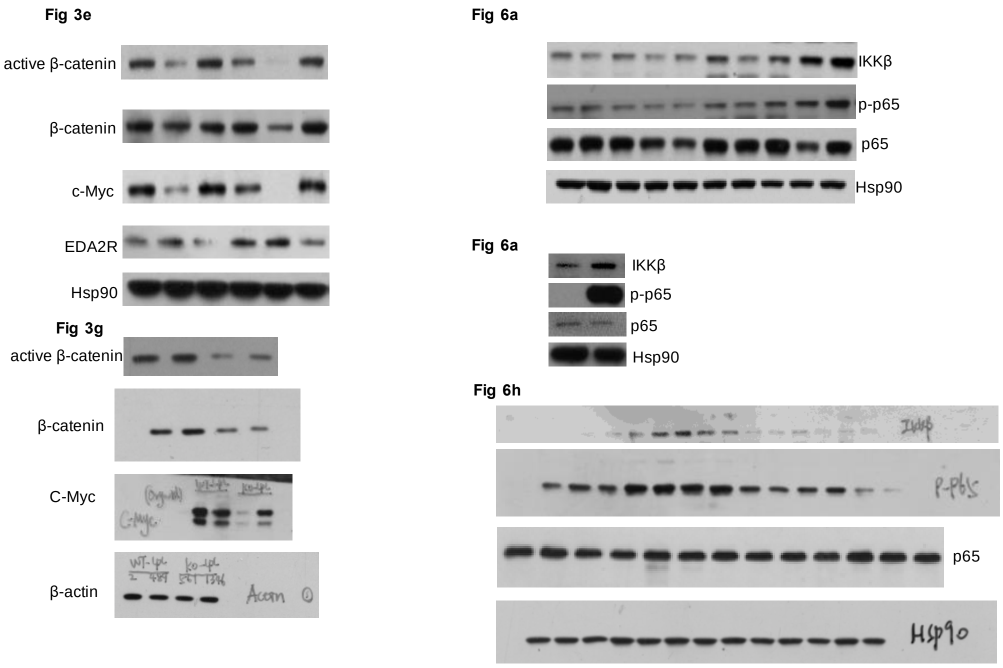

Supplementary Figure 10 Blot images shown in the study.

Supplementary Table 1 Human Samples Information

Clinical characteristics of patients with active IBD and control donors

Colon Tissues Samples	Normal (N=12)	Active UC (N=13)	Active CD (N=18)
Age (years)	53±11.91	41.79±17.96	34.17±12.4
Gender			
Male	8	6	12
Female	4	7	6
Current therapy			
5-aminosalicylates		7	7
Immuosuppressants		4	8
Biologics		1	3
Nutritional therapy		4	8
Disease extent (UC)			
E1		3	
E2		5	
E3		5	
Disease location (CD)			
L1			5
L2			2
L3			10
L4			1
CRP (mg/L)		19.7±17.25	25.4±18.37

Normal: individuals without IBD; CD: Crohn’s disease; UC: ulcerative colitis. Where indicated, data are presented as mean±S.D.

Human Sera Samples	Normal (N=20)	Active UC (N=33)	Active CD (N=22)
Age (years)	43.1±8.27	46.8±18.09	39.2±7.46
Gender			
Male	12	18	12
Female	8	15	10
Current therapy			
5-aminosalicylates		21	7
Immuosuppressants		6	8
Biologics		9	3
Nutritional therapy		15	8
Disease extent (UC)			
E1		15	
E2		3	
E3		15	
Disease location (CD)			
L1			9
L2			11
L3			2
L4			0
CRP (mg/L)		26.9±20.78	33.0±26.56

Normal: individuals without IBD; CD: Crohn’s disease; UC: ulcerative colitis. Where indicated, data are presented as mean±S.D.

\

Supplementary Table 2 mouse genotyping PCR primer sequences

Primer	sequence 5'-3'
Primer 1	GGTCGCTTCTCATCACCCAC
Primer 2	AGTAGAAGGTGGCGCGAAGG
Primer 3	GGGAAGCAGCCAATGATTTG

Supplementary Table 3 qRT-PCR primer sequences

Gene	Forward Primer 5'-3'	Reverse Primer 5'-3'
mmiR-494-3p	CGGGCTGAAACATACACGGG	CAGCCACAAAAGAGCACAAT
mmiR-182-5p	CGGGCTTTGGCAATGGTAGAACT	CAGCCACAAAAGAGCACAAT
mmiR-200b-3p	CGGGCTAATACTGCCTGGTA	CAGCCACAAAAGAGCACAAT
mmiR-200b-5p	CGGGCCATCTTACTGGGCAG	CAGCCACAAAAGAGCACAAT
mmiR-200a-3p	CGGGCTAACACTGTCTGGTA	CAGCCACAAAAGAGCACAAT
mmiR-192-3p	CGGGCCTGCCAATTCCATAG	CAGCCACAAAAGAGCACAAT
mmiR-194-5p	CGGGCTGTAACAGCAACTCC	CAGCCACAAAAGAGCACAAT
mmiR-141-3p	CGGGCTAACACTGTCTGGTA	CAGCCACAAAAGAGCACAAT
mmiR-429-3p	CGGGCTAATACTGTCTGGTA	CAGCCACAAAAGAGCACAAT
mmiR-215-5p	CGGGCATGACCTATGATTTG	CAGCCACAAAAGAGCACAAT
mmiR-494-5p	AGGUUGUCCGUGUUGUCUUCUC	CAGCCACAAAAGAGCACAAT
U6	AACGCTTCACGAATTTGCGT	AACGCTTCACGAATTTGCGT
cel-miR-39	CGGGCTCACCGGGTGTAAAT	CAGCCACAAAAGAGCACAAT
mArg1	ACCTGGCCTTTGTTGATGTCCCTA	AGAGATGCTTCCAACTGCCAGACT
mCOX2	GATGCTCTTCCGAGCTGTG	GGATTGGAACAGCAAGGATTT
mFizz1	ACTGCCTGTGCTTACTCGTTGACT	AAAGCTGGGTTCTCCACCTCTTCA
mIFNγ	GCCACGGCACAGTCATTGA	TGCTGATGGCCTGATTGTCTT
miNOS	TCCTGGAGGAAGTGGGCCGAAG	CCTCCACGGGCCCGGTACTC
mIL-1β	GCAACTGTTCCTGAACTCAACT	ATCTTTTGGGGTCCGTCAACT
mIL-6	GATGGATGCTACCAAACTGGAT	CCAGGTAGCTATGGTACTCCAGA
mIL-10	GCTCTTACTGACTGGCATGAG	CGCAGCTCTAGGAGCATGTG
mIL-12β	TGGTTTGCCATCGTTTTGCTG	ACAGGTGAGGTTCACTGTTTCT
mTNFα	CCCTCACACTCAGATCATCTTCT	GCTACGACGTGGGCTACAG
mTGFβ	CTCCCGTGGCTTCTAGTGC	GCCTTAGTTTGGACAGGATCTG
mYm1	CACCATGGCCAAGCTCATTCTTGT	TATTGGCCTGTCCTTAGCCCAACT
mCCL2	TTAAAAACCTGGATCGGAACCAA	GCATTAGCTTCAGATTTACGGGT
mCCL5	GCTGCTTTGCCTACCTCTCC	TCGAGTGACAAACACGACTGC
mCCL6	GCTGGCCTCATACAAGAAATGG	GCTTAGGCACCTCTGAACTCTC
mCXCL1	AGACTCCAGCCACACTCCAA	TGACAGCGCAGCTCATTG
mCXCL2	CCTGGTTCAGAAAATCATCCA	CTTCCGTTGAGGGACAGC
mCXCL10	CCAAGTGCTGCCGTCATTTTC	GGCTCGCAGGGATGATTTCAA
mCXCL12	TGCATCAGTGACGGTAAACCA	TTCTTCAGCCGTGCAACAATC
mCSF1	ATGAGCAGGAGTATTGCCAAGG	TCCATTCCCAATCATGTGGCTA
mCSF2	GGCCTTGGAAGCATGTAGAGG	GGAGAACTCGTTAGAGACGACTT
mEDA-A2	TATAGTCAGGTCTACTACAT	CTCAATGCTGCGGGTGCACTGCA

mEDA2R	CACACTGCATAGTCTGCCCTC	GCCTTCTGGACCCGATTGA
mLEF1	TGTTTATCCCATCACGGGTGG	CATGGAAGTGTCGCCTGACAG
mMyc	ATGCCCCTCAACGTGAACTTC	CGCAACATAGGATGGAGAGCA
mFos	CGGGTTTCAACGCCGACTA	TTGGCACTAGAGACGGACAGA
mcyclinD1	GCGTACCCTGACACCAATCTC	CTCCTCTTCGCACTTCTGCTC
mMMP7	CTGCCACTGTCCCAGGAAG	GGGAGAGTTTTCCAGTCATGG
mLgr5	CCTACTCGAAGACTTACCCAGT	GCATTGGGGTGAATGATAGCA
mBMI1	ATCCCCACTTAATGTGTGTCCT	CTTGCTGGTCTCCAAGTAACG
mActin	GGCTGTATTCCCCTCCATCG	CCAGTTGGTAACAATGCCATGT
mGAPDH	AGGTCGGTGTGAACGGATTTG	TGTAGACCATGTAGTTGAGGTCA
hGAPDH	CTGGGCTACACTGAGCACC	AAGTGGTCGTTGAGGGCAATG
hIL-1β	ATGATGGCTTATTACAGTGGCAA	GTCGGAGATTCGTAGCTGGA
hIL-6	ACTCACCTCTTCAGAACGAATTG	CCATCTTTGGAAGGTTCAGGTTG
hIKKβ	GTCTTTGCACATCATTCGTGGG	GTGCCGAAGCTCCAGTAGTC

Supplementary Table 4 RT-PCR primer sequences

Gene	Sequence 5’-3’
mmiR-494-3p RT	CCTGTTGTCTCCAGCCACAAAAGAGCACAATATTTCAGGAGACAACAGGGAGGTTT
mmiR-182-5p RT	CCTGTTGTCTCCAGCCACAAAAGAGCACAATATTTCAGGAGACAACAGGCGGTGTG
mmiR-200b-3p RT	CCTGTTGTCTCCAGCCACAAAAGAGCACAATATTTCAGGAGACAACAGGTCATCAT
mmiR-200b-5p RT	CCTGTTGTCTCCAGCCACAAAAGAGCACAATATTTCAGGAGACAACAGGTCCAATG
mmiR-200a-3p RT	CCTGTTGTCTCCAGCCACAAAAGAGCACAATATTTCAGGAGACAACAGGACATCGT
mmiR-192-3p RT	CCTGTTGTCTCCAGCCACAAAAGAGCACAATATTTCAGGAGACAACAGGCTGTGAC
mmiR-194-5p RT	CCTGTTGTCTCCAGCCACAAAAGAGCACAATATTTCAGGAGACAACAGGTCCACAT
mmiR-141-3p RT	CCTGTTGTCTCCAGCCACAAAAGAGCACAATATTTCAGGAGACAACAGGCCATCTT
mmiR-429-3p RT	CCTGTTGTCTCCAGCCACAAAAGAGCACAATATTTCAGGAGACAACAGGACGGCAT
mmiR-215-5p RT	CCTGTTGTCTCCAGCCACAAAAGAGCACAATATTTCAGGAGACAACAGGGTCTGTC
mmiR-494-5p RT	CCTGTTGTCTCCAGCCACAAAAGAGCACAATATTTCAGGAGACAACAGGGAGAAGA
U6 RT	AACGCTTCACGAATTTGCGT
Cel-miR-39 RT	CCTGTTGTCTCCAGCCACAAAAGAGCACAATATTTCAGGAGACAACAGGCAAGCTG

Supplementary Methods

Exosome isolation. The exosome isolation assay was performed as described previously[5]. In our study, human serum (1 mL/sample) was used to isolate exosomes and the isolated exosomes then were used to measure miR-494-3p expression.

RNA-FISH Assay. The colonic tissues from the indicated mice were removed, washed with PBS (pH 7.4), and then immediately fixed in 4% PFA in PBS (pH 7.4) (pre-treated with DEPC). The fixed tissues were embedded in paraffin and sectioned at 5-μm thicknesses with an Ultracut microtome (Leica, Bannockburn, IL, USA). The sections were dehydrated, digested with Protein K (20 μg/mL) for 25 min, pre-hybridized for 1 hour, and hybridized with mmu-mir-494-3p probe overnight at 37 °C. The mir-494-3p probe consisted of a FAM-labeled oligo nucleotide with the sequence 5'-FAM-GAGGTTTCCCGTGTATGTTTCA-FAM-3' (Guangzhou Ribobio Co., Ltd., China). Washed sections were incubated with DAPI staining buffer for 8 min and then mounted. All sections were examined and photographed with an Olympus IX81 (miR-494-3p-FAM: 488nm, DAPI: 405nm).

Transmission electron microscopy (TEM). Colon tissues were isolated from 8-week-old male mice that had been provided with free access to 3% DSS in drinking water for 7 days. Following collection, tissues were washed with PBS (pH 7.4), then prepared and treated as described previously[6].

RNA-seq and microRNA Microarray. Cells or tissues from the indicated mice were extracted using Trizol reagent (Takara) and RNAs were isolated using Qiagen RNAeasy kits. The resulting samples were reverse transcribed to yield cDNA, which then was assayed by Hiseq RNA-seq or microRNA microarray. In this analysis, the RNA Integrity Numbers (RINs) of all samples exceeded 8 (scale 0–10). Hiseq RNA-seq was performed by GBI Tech Solution, and transcriptomic reads were mapped to a reference genome using Hisat2 (version: 2.0.4) and STAR v2.7 with NCBI RefSeq genes. Then the RSEM, ggplots, Rtsne, gdata, gsva, prcomp, and GO starts software packages were used to quantify gene expression levels and to further analyze, and results were filtered for significant changes in expression according to the following criteria: fold-change ≥ 2, P value ≤ 0.05, and false discovery rate (FDR) threshold of 0.001. For the microRNA microarray, the assessment and analysis were performed as described previously[22]. In our study, RINs of all samples exceeded 8 (scale 0–10). Differentially expressed genes were filtered by both P value ≤ 0.05 and fold-change ≥ 2.

For IEC RNA-seq, IECs from C57BL/6 mice (3- to 4-week-old males) were cultured for 4-5 days in 6-well plates and then transfected with miR-494 mimic (OE) and control mimic (control) for 24 hours, followed by treatment with 1 μg/mL LPS for another 24 hours. For colonic crypt RNA-seq and microRNA microarray, whole colons were removed from mice that had been provided with free access to with normal drinking water or 3% DSS in drinking water for 7 days; crypts were then isolated according to the "Colonic Organoid Culture" protocol. These cells were

directly ground in Trizol and used to isolate RNA.

Supplementary References

1. Coburn L.A., et al. L-arginine supplementation improves responses to injury and inflammation in dextran sulfate sodium colitis. *PLoS One*. **7**, e33546 (2012).
2. Pekow J., et al. miR-193a-3p is a Key Tumor Suppressor in Ulcerative Colitis-Associated Colon Cancer and Promotes Carcinogenesis through Upregulation of IL17RD. *Clin Cancer Res*. **23**, 5281-5291 (2017).
3. Fang K., et al. Temporal genome wide expression profiling of DSS colitis reveals novel inflammatory and angiogenesis genes similar to ulcerative colitis. *Physiol Genomics*. **43**, 43-56 (2011).
4. Noble C.L., et al. Regional variation in gene expression in the healthy colon is dysregulated in ulcerative colitis. *Gut*. **57**, 1398-405 (2008).
5. Thomou T., et al. Adipose-derived circulating miRNAs regulate gene expression in other tissues. *Nature*. **542**, 450-455 (2017).
6. Luo H., Guo P., Zhou Q. Role of TLR4/NF-Kb in damage to intestinal mucosa barrier function and bacterial translocation in rats exposed to hypoxia. *PLoS One*. **7**, e46291 (2012).
