# Supplementary material for: Macrophage-derived EDA-A2 inhibits intestinal stem cells by targeting miR-494/EDA2R/β-catenin signaling in mice
Source: Commun Biol. 2021 Feb 16;4:213. doi: 10.1038/s42003-021-01730-0 (PMC7887198; doi:10.1038/s42003-021-01730-0)
Supplement: Supplementary file 2 — Description of Additional Supplementary Files [file 42003_2021_1730_MOESM2_ESM.pdf]

Description of Additional Supplementary Files

File Name: Supplementary Data 1
Description: The source data underlying the graphs and charts presented in the main figures.
