# Supplementary material for: Macrophage-derived EDA-A2 inhibits intestinal stem cells by targeting miR-494/EDA2R/β-catenin signaling in mice
Source: Commun Biol. 2021 Feb 16;4:213. doi: 10.1038/s42003-021-01730-0 (PMC7887198; doi:10.1038/s42003-021-01730-0)
Supplement: Supplementary file 4 — Reporting Summary [file 42003_2021_1730_MOESM4_ESM.pdf]

nature research

Corresponding author(s): Lixing Zhan, Zhanju Liu

Last updated by author(s): Aug 27, 2020

Reporting Summary

Nature Research wishes to improve the reproducibility of the work that we publish. This form provides structure for consistency and transparency in reporting. For further information on Nature Research policies, seeAuthors & Referees and theEditorial Policy Checklist .

Statistics

For all statistical analyses, confirm that the following items are present in the figure legend, table legend, main text, or Methods section.

n/a	Confirmed	
☐	☒	The exact sample size (*n*) for each experimental group/condition, given as a discrete number and unit of measurement
☐	☒	A statement on whether measurements were taken from distinct samples or whether the same sample was measured repeatedly
☐	☒	The statistical test(s) used AND whether they are one- or two-sided<br>*Only common tests should be described solely by name; describe more complex techniques in the Methods section.*
☒	☐	A description of all covariates tested
☒	☐	A description of any assumptions or corrections, such as tests of normality and adjustment for multiple comparisons
☐	☒	A full description of the statistical parameters including central tendency (e.g. means) or other basic estimates (e.g. regression coefficient) AND variation (e.g. standard deviation) or associated estimates of uncertainty (e.g. confidence intervals)
☐	☒	For null hypothesis testing, the test statistic (e.g. *F*, *t*, *r*) with confidence intervals, effect sizes, degrees of freedom and *P* value noted<br>*Give P values as exact values whenever suitable.*
☒	☐	For Bayesian analysis, information on the choice of priors and Markov chain Monte Carlo settings
☒	☐	For hierarchical and complex designs, identification of the appropriate level for tests and full reporting of outcomes
☒	☐	Estimates of effect sizes (e.g. Cohen's *d*, Pearson's *r*), indicating how they were calculated

Our web collection on statistics for biologists contains articles on many of the points above.

Software and code

Policy information about availability of computer code

Data collection	Confocal Images were captured by Olympus FV10-ASW 3.1. Images of tissues were captured by Qcapture Suite. Imgaes of organoids and cells were captured by XV Image Processing.<br>ABI 7900 SDS 2.4 software was used for qPCR acquisition.<br>BD FACSDiva software was used for flow cytometry.<br>Correlation analysis was conducted in R and microsoft excel (2007). Heatmaps generated with the pheatmap package.<br>ImageJ 1.44P was used for quantification of positive cells.
Data analysis	GraphPad prism (version 6.01) (GraphPad Software, Inc.) was used to analyze the data.<br>The pair-ended reads were mapped to the mouse genome (UCSC mm10) using STAR v2.7 with NCBI RefSeq genes as the reference.<br>Further analyses were performed using R v3.3, including packages ggplot2, gplots, Rtsne, gdata, gsva, prcomp, and GOstats were used.<br>FACS data were analyzed using FlowJo v10.0 and FlowJo v 7.6.1.<br>Please refer to methods section for details.

For manuscripts utilizing custom algorithms or software that are central to the research but not yet described in published literature, software must be made available to editors/reviewers. We strongly encourage code deposition in a community repository (e.g. GitHub). See the Nature Research guidelines for submitting code & software for further information.

Data

Policy information about availability of data

All manuscripts must include a data availability statement. This statement should provide the following information, where applicable:

- Accession codes, unique identifiers, or web links for publicly available datasets
- A list of figures that have associated raw data
- A description of any restrictions on data availability

All the sequencing data in this study have been deposited in the NCBI with the accession numbers (GSE137889, GSE137890, and GSE137892), and have been open

to public. Other secondary datasets used in this study include: GSE34874, GSE68306, GSE22307, GSE11223. All other data supporting the findings of this study are available from the corresponding author on reasonable request.

Field-specific reporting

Please select the one below that is the best fit for your research. If you are not sure, read the appropriate sections before making your selection.

- [x] Life sciences
- [ ] Behavioural & social sciences
- [ ] Ecological, evolutionary & environmental sciences

For a reference copy of the document with all sections, see nature.com/documents/nr-reporting-summary-flat.pdf

Life sciences study design

All studies must disclose on these points even when the disclosure is negative.

Sample size	No statistical method was used to predetermine sample size. Sample size was chosen based on previous experience and standards in the field. The sample sizes for in vitro (n>=3) and in vivo (n >=4-6) are typical of the field. All Sample sizes are listed in the corresponding figure legend
Data exclusions	No data were excluded from the analyses.
Replication	All of the experiments were repeated at least 3 times and the findings were reliably reproduced.
Randomization	All the samples, organisms and participants were randomly allocated into different experimental groups.
Blinding	All samples were not blinded to the investigators. Bind experiments were not necessary as all measurements were objective, but no subjective assessments were made.

Reporting for specific materials, systems and methods

We require information from authors about some types of materials, experimental systems and methods used in many studies. Here, indicate whether each material, system or method listed is relevant to your study. If you are not sure if a list item applies to your research, read the appropriate section before selecting a response.

Materials & experimental systems

n/a	Involved in the study	
[ ]	[x]	Antibodies
[ ]	[x]	Eukaryotic cell lines
[x]	[ ]	Palaeontology
[ ]	[x]	Animals and other organisms
[ ]	[x]	Human research participants
[x]	[ ]	Clinical data

Methods

n/a	Involved in the study	
[x]	[ ]	ChIP-seq
[ ]	[x]	Flow cytometry
[x]	[ ]	MRI-based neuroimaging

Antibodies

Antibodies used

1. Immunohistochemistry (IHC) stanining
Rabbit Anti-Ki67 (1:200, #NBP1-40684, Novus)
2. Immunofluorescence (IF) staining
Rabbit Anti-F4/80 (1:400, #30325, Cell Signaling Technology; Boston, MA, USA);
Rabbit Anti-Ki67 (1:200, #ab15580, Abcam, Cambridge, UK);
The secondary antibodies: goat anti-rabbit IgG highly cross-adsorbed secondary antibody, Alexa Fluor Plus 488 (1:250, #A32731, ThermoFisher Scientific, Waltham, MA) or 568 (1:250, #A-11011, ThermoFisher Scientific)
3. Flow cytometry
FITC-conjugated anti-CD11b (1:200, #11-0112-82, eBioscience, San Diego, CA), APC-conjugated anti-CD11c (1:200, #17-0114-81, eBioscience), PE-cyanine7-conjugated anti-F4/80 (1:200, #25-4801-82, eBioscience), PE-conjugated anti-CD206 (1:200, #12-2061-80, eBioscience), and PE-conjugated anti-Ly6G (1:1500, #12-5931-81, eBioscience); for T cell analyses, cells were incubated with the following antibodies: APC-conjugated anti-CD3 (1:200, #17-0032-82, eBioscience), FITC-conjugated anti-CD4 (1:200, #11-0042-81, eBioscience), and PE-conjugated anti-CD8 (1:200, #12-0081-81, eBioscience); for B cell and NK cell analyses, cells were incubated with the following antibodies: FITC-conjugated anti-CD19 (1:200, #11-0193-81, eBioscience), and PE-conjugated anti-NK1.1 (1:200, #12-5941-81, eBioscience).
4. Western blot Analysis
Rabbit Anti-IKKβ (1:1000, #8943, Cell Signaling Technology; Boston, MA, USA);
Rabbit Anti- p-p65 (1:1000, #3033, Cell Signaling Technology);
Rabbit Anti- p65(1:1000, #8242, Cell Signaling Technology);

Rabbit Anti- Hsp90(1:1000, #4877, Cell Signaling Technology);
Rabbit Anti-β-catenin(1:1000, #8480, Cell Signaling Technology);
Mouse Anti-c-Myc (1:1000, #sc-40, Santa Cruz Biotechnology; Santa Cruz, CA, USA);
Mouse Anti-β-actin (1:1000, #30101ES10, Yeasen Biotechnology Co., Ltd.; Shanghai, China);
Mouse Anti-EDA2R (1:1000, #ab167224, Abcam, Cambridge, UK).

Validation

All antibodies were from commercial source and validated by the manufacturer for the species and application used in this study.
Rabbit Anti-Ki67 (#NBP1-40684, Novus Biological), validation: https://www.novusbio.com/products/ki67-mki67-antibody-epr3611_nbp1-40684
Rabbit Anti-F4/80 (#30325, Cell Signaling Technology), validation; https://www.cst-c.com.cn/products/primary-antibodies/f4-80-d4c8v-xp-rabbit-mab/30325
Rabbit Anti-Ki67 (#ab15580, Abcam), validation: https://www.abcam.cn/ki67-antibody-ab15580.html
goat anti-rabbit IgG highly cross-adsorbed secondary antibody, Alexa Fluor Plus 488 (#A32731, ThermoFisher Scientific, Waltham, MA) or 568 (#A-11011, ThermoFisher Scientific), validation: https://www.thermofisher.com/cn/zh/antibody/product/Goat-anti-Rabbit-IgG-H-L-Highly-Cross-Adsorbed-Secondary-Antibody-Polyclonal/A32731 https://www.thermofisher.com/cn/zh/antibody/product/Goat-anti-Rabbit-IgG-H-L-Cross-Adsorbed-Secondary-Antibody-Polyclonal/A-11011
FITC-conjugated anti-CD11b (#11-0112-82, ebioscience), validation: https://www.thermofisher.com/cn/zh/antibody/product/CD11b-Antibody-clone-M1-70-Monoclonal/11-0112-82
APC-conjugated anti-CD11c (#17-0114-81, eBioscience), validation: https://www.thermofisher.com/cn/zh/antibody/product/CD11c-Antibody-clone-N418-Monoclonal/17-0114-81
PE-cyanine7-conjugated anti-F4/80 (#25-4801-82, eBioscience), validation: https://www.thermofisher.com/cn/zh/antibody/product/F4-80-Antibody-clone-BM8-Monoclonal/25-4801-82
PE-conjugated anti-CD206 (#12-2061-80, eBioscience), validation: https://www.thermofisher.com/cn/zh/antibody/product/CD206-MMR-Antibody-clone-MR6F3-Monoclonal/12-2061-80
PE-conjugated anti-Ly6G (#12-5931-81, eBioscience), validation: https://www.thermofisher.com/cn/zh/antibody/product/Ly-6G-Ly-6C-Antibody-clone-RB6-8C5-Monoclonal/12-5931-81
APC-conjugated anti-CD3 (#17-0032-82, eBioscience), validation: https://www.thermofisher.com/cn/zh/antibody/product/CD3-Antibody-clone-17A2-Monoclonal/17-0032-82
FITC-conjugated anti-CD4 (#11-0042-81, eBioscience), validation: https://www.thermofisher.com/cn/zh/antibody/product/CD4-Antibody-clone-RM4-5-Monoclonal/11-0042-81
PE-conjugated anti-CD8 (#12-0081-81, eBioscience), validation: https://www.thermofisher.com/cn/zh/antibody/product/CD8a-Antibody-clone-53-6-7-Monoclonal/12-0081-81
FITC-conjugated anti-CD19 (#11-0193-81, eBioscience), validation: https://www.thermofisher.com/cn/zh/antibody/product/CD19-Antibody-clone-eBio1D3-1D3-Monoclonal/11-0193-81
PE-conjugated anti-NK1.1 (#12-5941-81, eBioscience), validation: https://www.thermofisher.com/cn/zh/antibody/product/NK1-1-Antibody-clone-PK136-Monoclonal/12-5941-81
Rabbit Anti-IKKβ (#8943, Cell Signaling Technology; Boston, MA, USA), validation: https://www.cst-c.com.cn/products/primary-antibodies/ikkb-d30c6-rabbit-mab/8943?site-search-type=Products
Rabbit Anti- p-p65 (#3033, Cell Signaling Technology), validation: https://www.cst-c.com.cn/products/primary-antibodies/phospho-nf-kb-p65-ser536-93h1-rabbit-mab/3033?site-search-type=Products
Rabbit Anti- p65(#8242, Cell Signaling Technology, validation: https://www.cst-c.com.cn/products/primary-antibodies/nf-kb-p65-d14e12-xp-rabbit-mab/8242?site-search-type=Products
Rabbit Anti- Hsp90(#4877, Cell Signaling Technology), validation: https://www.cst-c.com.cn/products/primary-antibodies/hsp90-antibody/4874?site-search-type=Products
Rabbit Anti-β-catenin(#8480, Cell Signaling Technology), validation: https://www.cst-c.com.cn/products/primary-antibodies/b-catenin-d10a8-xp-rabbit-mab/8480?site-search-type=Products
Mouse Anti-β-actin (#30101ES10, Yeasen Biotechnology Co., Ltd.), validation: https://www.yeasen.com/products/detail/877
Mouse Anti-c-Myc (#sc-40, Santa Cruz Biotechnology), validation: https://www.scbt.com/zh/p/c-myc-antibody-9e10?requestFrom=search
Mouse Anti-EDA2R (#ab167224, Abcame), validation: https://www.abcam.cn/eda2rxedar-antibody-ab167224.html

Eukaryotic cell lines

Policy information about cell lines

Cell line source(s)

We used mouse macrophage RAW264.7 (ATCC, Cat# TIB-71) and human embryonic kidney (HEK) 293 cells (ATCC, Cat# CRL-1573) in our study.

Authentication

None of the cell lines were authenticated

Mycoplasma contamination

We have tested the RAW264.7 and 293 cells for mycoplasma contamination and the results were negative.

Commonly misidentified lines
(See ICLAC register)

No commonly misidentified lines were used.

Animals and other organisms

Policy information about studies involving animals; ARRIVE guidelines recommended for reporting animal research

Laboratory animals	To generate miR-494-/- mice, the Sanger MirKO ES cell line miR494 (the whole locus of miR-494 is knockout in this cell line) was purchased from The Jackson Laboratory (Bar Harbor, ME), and microinjected into C57BL/6 mice at Shanghai Model Organisms (Shanghai, China); resulting male chimeric mice were crossed with female C57BL/6 to generate heterozygous mice, which then were crossed to generate miR-494+/+ (wild-type, WT) and miR-494-/- (knockout, KO) mice. For Dextran Sulfate Sodium (DSS)-induced colitis model, clodronate-mediated macrophage deletion model, IKKβ knockdownassay and DSS-induced colitis with miR-494-3p agomir treatment model, we used age- (8 to 12-week-old) and gender- (male) matched mice. Colonic crypts, lamina propria lymphocytes and BMDM were isolated from age-(8 to 10-week-old) matched male mice and IECs were isolated from male mice (3 to 4-week-old). For BMDM Chimeras Assay, 6 week-old male C57BL/6 (CD45.1) and miR-494-/- (CD45.2) mice were irradiated and 8 week-old male C57BL/6 or miR-494+/+ or miR-494-/- mice were used to isolate BMDM.
Wild animals	Our study did not involve wild animals.
Field-collected samples	Our study did not involve samples collected from the field.
Ethics oversight	All animal experiments were performed in accordance with a protocol approved by the Institutional Animal Care and Use Committee at the Shanghai Institutes for Biological Sciences, Chinese Academy of Sciences (Shanghai, China).

Note that full information on the approval of the study protocol must also be provided in the manuscript.

Human research participants

Policy information about studies involving human research participants

Population characteristics	Colon tissues and sera were collected from IBD patients who had been pathologically confirmed to have active UC (A-UC) or CD (A-CD). Colon tissues were taken at the time of endoscopy and uninflamed tissues from the respective patient were used as controls. Sera from healthy volunteers and active IBD patients were collected in the course of blood collection for clinical examination. The age at presentation varied from 20 to 67 years. The gender distribution was about 1:1. The detail characteristics (age, gender, current therapy, disease extent (UC), disease location (CD), and CRP) were included in the “Table S4” section of the supplementary materials.
Recruitment	All of patients were recruited from the Shanghai Tenth People’s Hospital of Tongji University (Shanghai, China).Colon tissues were taken at the time of endoscopy and uninflamed tissues from the respective patient were used as controls. Sera from healthy volunteers and active IBD patients were collected in the course of blood collection for clinical examination. No bias was identified. Before the study, we obtained written informed consent from all participants.
Ethics oversight	The use of human specimens was approved by the Ethics Review Board of the Shanghai Tenth People’s Hospital (Tongji University).

Note that full information on the approval of the study protocol must also be provided in the manuscript.

Flow Cytometry

Plots

Confirm that:

- [x] The axis labels state the marker and fluorochrome used (e.g. CD4-FITC).
- [x] The axis scales are clearly visible. Include numbers along axes only for bottom left plot of group (a 'group' is an analysis of identical markers).
- [x] All plots are contour plots with outliers or pseudocolor plots.
- [x] A numerical value for number of cells or percentage (with statistics) is provided.

Methodology

Sample preparation	Lamina Propria Lymphocytes (LPL)<br>For isolation of LPL, the whole colon was removed, cut into fragments of about 1 cm in length, and rinsed with HBSS buffer. The rinsed colon fragments then were gently shaken (250 rpm) for 30 min at 37 °C in HBSS buffer supplemented with 30 mM EDTA (sigma, Darmstadt, Germany), 1 mM DTT (sigma), and 5% (vol/vol) FBS. After sedimentation by standing, the supernatant was discarded and the remaining colon tissues were further cut into smaller pieces and incubated with digestive solution [RPMI-1640 medium (Gibco) supplemented with 200 U/mL collagenase VIII (sigma), 150 μg/mL DNAase I (sigma), 5% (vol/vol) FBS, and 1% (vol/vol) penicillin and streptomycin] for 1 hour at 37 °C. After digestion, the supernatant was centrifuged at 450xg for 5 min and Percoll (40%/80%; GE Healthcare) was used to isolate LPLs. The LPLs then were subjected to flow cytometry.<br><br>splenic Immune Cells<br>For isolation of immune cells from the spleen, spleen was crushed and passed through a 40-μm filter to obtain a single-cell

suspension. After centrifugation at 300xg for 5 min, cells were resuspended in red blood cell lysing buffer for 5 min and then again passed through a 40-μm filter. The resulting suspension was subjected to another round of centrifugation at 300xg for 5 min and the resulting supernatant was discarded. The remaining cells were washed 2 times with PBS (pH=7.4) and then subjected to flow cytometry.

Instrument	All data were acquired using BD FACSDiva software. FACS sorting and analyses were performed using FACSAria II (BD Biosciences)
Software	All data were analyzed with Flow Jo v10.0 and Flow Jo v 7.6.1.
Cell population abundance	> 85% for downstream applications.
Gating strategy	colonic LP macrophage: gated for CD11b+CD11C-F4/80+ cells; M1 macrophage: gated for CD11b+CD11C-F4/80+CD206- cells; M2 macrophage: gated for CD11b+CD11C-F4/80+CD206+ cells<br>LP neutrophils: gated for CD11b+Gr1hi<br>splenic CD4 cells: gated for CD3+CD4+; splenic CD8 cells: gated for CD3+CD8+; splenic B cells: gated for CD19+NK1.1-; splenic NK1.1 cells: gated for CD19-NK1.1+

☒ Tick this box to confirm that a figure exemplifying the gating strategy is provided in the Supplementary Information.
